# Muscle mass, strength, and physical performance predicting activities of daily living: a meta‐analysis

**DOI:** 10.1002/jcsm.12502

**Published:** 2019-12-01

**Authors:** Daniel X.M. Wang, Jessica Yao, Yasar Zirek, Esmee M. Reijnierse, Andrea B. Maier

**Affiliations:** ^1^ Department of Medicine and Aged Care, @AgeMelbourne The University of Melbourne, The Royal Melbourne Hospital Parkville VIC Australia; ^2^ Department of Human Movement Sciences, @AgeAmsterdam Vrije Universiteit Amsterdam, Amsterdam Movement Sciences Amsterdam The Netherlands

**Keywords:** Muscle mass, Muscle strength, Handgrip strength, Physical performance, Activities of daily living, Aged

## Abstract

**Background**

Activities of daily living (ADLs) and instrumental activities of daily living (IADLs) are essential for independent living and are predictors of morbidity and mortality in older populations. Older adults who are dependent in ADLs and IADLs are also more likely to have poor muscle measures defined as low muscle mass, muscle strength, and physical performance, which further limit their ability to perform activities. The aim of this systematic review and meta‐analysis was to determine if muscle measures are predictive of ADL and IADL in older populations.

**Methods**

A systematic search was conducted using four databases (MEDLINE, EMBASE, Cochrane, and CINAHL) from date of inception to 7 June 2018. Longitudinal cohorts were included that reported baseline muscle measures defined by muscle mass, muscle strength, and physical performance in conjunction with prospective ADL or IADL in participants aged 65 years and older at follow‐up. Meta‐analyses were conducted using a random effect model.

**Results**

Of the 7760 articles screened, 83 articles were included for the systematic review and involved a total of 108 428 (54.8% female) participants with a follow‐up duration ranging from 11 days to 25 years. Low muscle mass was positively associated with ADL dependency in 5/9 articles and 5/5 for IADL dependency. Low muscle strength was associated with ADL dependency in 22/34 articles and IADL dependency in 8/9 articles. Low physical performance was associated with ADL dependency in 37/49 articles and with IADL dependency in 9/11 articles. Forty‐five articles were pooled into the meta‐analyses, 36 reported ADL, 11 reported IADL, and 2 reported ADL and IADL as a composite outcome. Low muscle mass was associated with worsening ADL (pooled odds ratio (95% confidence interval) 3.19 (1.29–7.92)) and worsening IADL (1.28 (1.02–1.61)). Low handgrip strength was associated with both worsening ADL and IADL (1.51 (1.34–1.70); 1.59 (1.04–2.31) respectively). Low scores on the short physical performance battery and gait speed were associated with worsening ADL (3.49 (2.47–4.92); 2.33 (1.58–3.44) respectively) and IADL (3.09 (1.06–8.98); 1.93 (1.69–2.21) respectively). Low one leg balance (2.74 (1.31–5.72)), timed up and go (3.41 (1.86–6.28)), and chair stand test time (1.90 (1.63–2.21)) were associated with worsening ADL.

**Conclusions**

Muscle measures at baseline are predictors of future ADL and IADL dependence in the older adult population.

## Introduction

Dependence in activities of daily living (ADLs), the basic tasks required of an individual to maintain their independence at home, is associated with increased risk of morbidity and mortality.^1^, ^2^ Individuals that are dependent in ADL are also likely to be dependent in instrumental activities of daily living (IADLs), the tasks required of an individual to maintain their independence in the community.^3^, ^4^, ^5^ The prevalence of ADL and IADL disability for at least one activity is 34.6% and 53.5%, respectively, in adults aged 65 years and older,^6^ and this prevalence increases with age.^7^ Those with lower muscle measures, defined by muscle mass, muscle strength, and physical performance,^8^ are more likely to be dependent in ADL and/or IADL.^9^, ^10^, ^11^ The more difficult tasks are for an individual, the more effort and demand they require relative to their muscle's maximum capacity.^12^

Older adults that develop ADL dependence are less likely to recover function, stressing the need for strategies that can prevent or delay the onset of ADL dependence.^13^ Higher muscle strength is protective against declining below the threshold where dependence in ADL and IADL occurs.^14^ In community‐dwelling older adults, physical performance measured by gait speed has been shown to be a strong predictor of ADL disability.^15^, ^16^ Similarly, low muscle mass, muscle strength, and gait speed have all been associated with an impaired ability to perform ADL and IADL.^17^, ^18^ Currently, there are no systematic review and/or meta‐analyses that quantifies the association between muscle mass, muscle strength, and physical performance as predictors of ADL and IADL dependence. By determining which muscle measures are predictive of ADL and IADL dependence allows for the identification of individuals at high risk of decline as well as the development and implementation of strategies that can prevent or delay the onset of dependence.

The aim of this systematic review and meta‐analysis was to determine if muscle mass, muscle strength, or physical performance are predictors of ADL and/or IADL at follow‐up in older populations.

## Methods

### Search strategy

This systematic review was conducted following the Preferred Reporting Items for Systematic Reviews and Meta‐analyses and registered with the PROSPERO International Prospective Register of Systematic Reviews (CRD42019125666).^19^ The following four electronic databases were screened for potential relevance from date of inception to 7 June 2018: MEDLINE, EMBASE, Cochrane Central Register of Controlled Trials, and Cumulative Index of Nursing and Allied Health Literature. The search strategy was developed in consultation with a senior tertiary librarian from The University of Melbourne, with expertise in research and search strategies. The following terms were used in the search strategy: ‘muscle mass’, ‘fat free mass’, ‘lean mass’, ‘muscle loss’/atrophy, ‘muscle strength’, ‘physical performance’/mobility/fitness/endurance, ‘activities of daily living’, ‘functional decline’/disability, and aged/elderly/older. The full search strategy can be found in Supporting Information, *Table* *S1*.

### Eligibility criteria

Inclusion criteria consisted of prospective longitudinal cohorts of older adults with a reported mean/median age of 65 years and older at follow‐up and reporting at least one of the following measurements: muscle mass, muscle strength, or physical performance in conjunction with a follow‐up outcome of ADL or IADL.

Exclusion criteria included cross‐sectional and case–control studies; anthropometric measurements as measures of muscle mass such as body mass index, hip‐waist ratio, waist or calf circumference measurements, and skin‐fold thickness; populations that suffered from cancer, muscular dystrophy, genetically inherited diseases, and HIV/AIDS; cohorts that received an intervention other than usual care or placebo; and non‐English articles. Articles including participants of the same cohort more than once were excluded in a hierarchical manner: (i) if there was no statistical analysis conducted regarding odds ratio (OR), hazards ratio, or relative risk and lacking data to calculate the OR; (ii) if their primary research question was not exploring the association between muscle measures with ADL or IADL; or (iii) an article had a smaller sample size.

### Article selection

Articles obtained through the search strategy had their title and abstract screened followed by full‐text screening for eligibility independently by two authors (D. W., Y. Z., or J. Y.) using Covidence (Covidence Systematic Review Software, Veritas Health Innovation, Melbourne, Australia). The decision made by authors was compared, and conflicts were settled by a third reviewer (E. M. R.).

### Data extraction and quality assessment

Data extraction was performed by two authors (D. W., Y. Z., or J. Y.). The following data were extracted from included articles: author, year, study/cohort, setting, country/region, demographical information [sample size, age (mean/median), and sex (% female)], and follow‐up duration. Muscle mass, muscle strength, physical performance, ADL, and IADL were extracted based on the measurement method, unit, and cut‐offs applied in analyses. Effect sizes were extracted from text, tables, or figures if not described elsewhere.

The quality of the included articles was assessed by a modified version of the Newcastle–Ottawa Scale (NOS) for cohort studies.^20^ Quality assessment was performed based on the following categories: selection, comparability, and outcome. Articles were deemed to be high quality with the following criteria: (i) the population was representative of the 65 years and older at follow‐up; (ii) in the case of a dichotomized sarcopenic cohort (low muscle mass, muscle strength, and physical performance), both cohorts were recruited from the same population; (iii) the technique used to measure muscle mass, muscle strength, or physical performance; (iv) the analysis was controlled for age and/or sex, as well as other factors; (v) ADL or IADL was measured with a validated method or a study designed questionnaire or survey; (vi) follow‐up duration was ≥3 months; and (vii) subjects were all followed up, accounted for or the number lost to follow‐up was unlikely to introduce bias (≤20%). The adapted version of the NOS can be found in Supporting Information, *Supplementary Material 2*. Studies above the median score (7/7 and 8/8) were the cut‐off point for articles of high quality.^21^

### Data synthesis and analysis

Effect sizes were extracted and reported where associations were made between baseline muscle measures and follow‐up ADL and/or IADL. Inclusion into the meta‐analysis required articles to report an OR, hazards ratio, relative risk, or if the article provided sufficient information to calculate an OR for the association between baseline muscle measures and follow‐up ADL and/or IADL. Forest plots were generated for the graphical representation of the meta‐analysis. A random effect model was adopted to account for differences between articles.^22^ Articles were presented in order from the shortest to longest follow‐up duration to determine if follow‐up duration impacts effect size, i.e. longer follow‐up duration showing greater effect size. Articles that reported sex‐stratified results or ADL and IADL were entered in the meta‐analysis separately. The least adjusted statistical model consisting of age and sex was used in the meta‐analysis, followed by the next least adjusted model then the unadjusted model. Heterogeneity was measured using the *I*‐squared (*I* ^2^) test. Low heterogeneity was defined as an *I* ^2^ ≤ 25%, moderate as 25–75%, and high as ≥75%.^23^ The *P*‐value for significance was set at <0.05, and the *P*‐value for a trend was set at 0.05 < *P* < 0.01. Meta‐analyses were performed separately according to the unit of gait speed, ADL, and IADL. The meta‐analysis was performed using Comprehensive Meta‐analysis (version 3.3; Biostat Inc., Englewood, NK).

## Results

**Figure 1:**
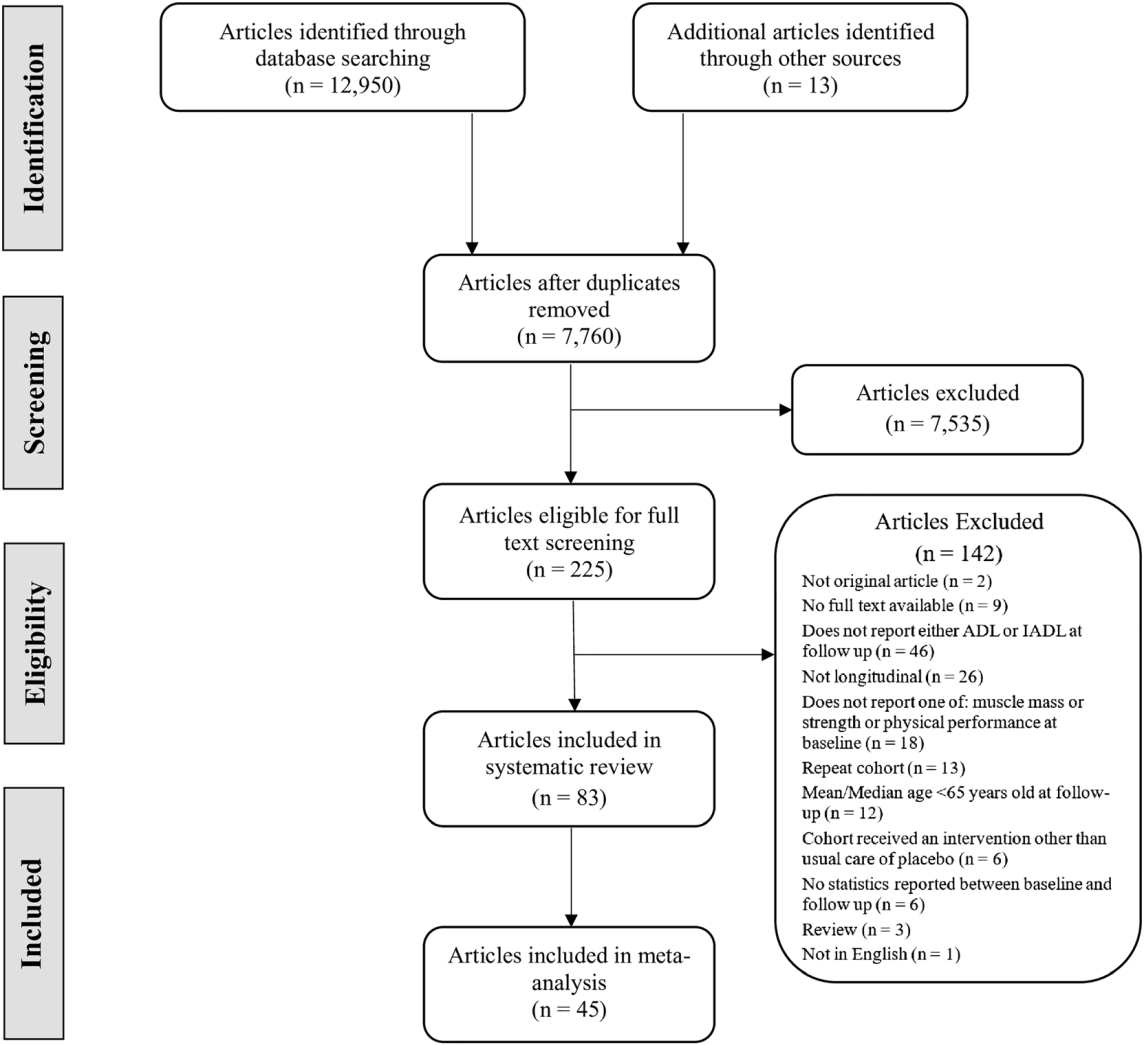
Preferred Reporting Items for Systematic Reviews and Meta‐analyses flow chart for the study selection process.

*Figure* 1 presents the Preferred Reporting Items for Systematic Reviews and Meta‐analyses flow diagram of selected articles. The four databases yielded 12 950 potential articles to which an additional 13 were added by snowballing. After removing duplicate articles, 7760 progressed to title and abstract screening. Of these, 7535 articles were excluded resulting in 225 articles for full‐text screening. Ultimately, 83 articles were included in the systematic review and 45 articles in the meta‐analysis.

### Characteristics of included articles

*Table* 1 presents the study characteristics of included articles. The majority of the articles were conducted on community‐dwelling participants (*n* = 65/83). The mean or median age of the study ranged from 54 to 86 years at baseline. The number of participants in each study ranged from 41 to 8000 and included a total of 108 428 (54.8% female) participants. Follow‐up duration ranged from 11 days to 25 years. ADL was measured in 73 articles that examined physical motor tasks, 30 of which had developed an adapted questionnaire to assess the motor component of ADL (Supporting Information, *Table* *S3*) and 21 articles using the Katz Index or a modified version. Of the articles reporting ADL, 63 articles used a dichotomous cut‐off reporting a worsening ADL score (≥1 points loss, i.e. more dependency) at follow‐up compared with using a continuous ADL score (*n* = 11). IADL was measured in 35 articles with the Lawton–Brody IADL scale being used in 14 articles, three of which were modified. A total of 26 articles included measures of both ADL and IADL.

**Table 1:** Characteristics of included studies and measured activities of daily living and instrumental activities of daily living

Study characteristics				Participants			FU	ADL		IADL		
First author (year) [ref]	Cohort name	Setting	Country	*N*	Age (years)	F (%)		Measure	Cut‐offs	Measure	Cut‐offs	
Abete (2017) 24	—	CD	ITA	907	81.3 ± 6.5	56.7	2 y	Katz	≥1 loss	—	—	
Al snih (2004) 25	HEPESE	CD	USA	2493	72.4 ± 6.2^a^	57.9	7 y	M Katz	≥1 loss	—	—	
Albert (2015) 26	SITE	CD	USA	375	78.9 ± 5.8	68.9	2 y	—	—	AMPS	Cont	
Alexandre (2012) 27	SABE	CD	BRA	1634	68.6 ± 0.4	57.1	6 y	M Katz	≥1 loss	—	—	
Amigues (2013) 28	EPIDOS	CD	FRA	975	79.9 ± 3.5	100.0	4 y	—	—	LB	≥1 loss	
Arnau (2016) 29	—	OP	ESP	252	81.7 ± 4.6	58.7	1 y	BI	≥10 loss	M LB	≥1 loss	
Artaud (2015) 30	3C	CD	FRA	3814	73.2 ± 4.6	60.9	11 y	M Katz	≥1 loss	LB	≥1 loss	
Basic (2017) 31	—	IP	AUS	1693	81.9 ± 7.5	61.5	11 d	M BI	≥1 loss	—	—	
Baumgartner (2004) 32	NMAPS	CD	USA	451	72.7 ± 6.3^a^	61.9	8 y	—	—	Own Q	≥1 loss	
Beauchamp (2015) 33	Boston RISE	PB	USA	360	76.6 ± 7.0	68.0	2 y	LLFDI‐FC	Cont	—	—	
Beloosesky (2009) 34	—	OP	ISR	93	81.2 ± 7.2	69.5	6 m	Own Q	Cont	—	—	
Bianchi (2015) 35	InCHIANTI	CD	ITA	538	77.1 ± 5.5	53.5	9 y	—	—	LB	≥1 loss	
Broadwin (2001) 36	—	CD	USA	1051	70.7^a^	60.3	4 y	Own Q	≥1 loss	—	—	
Carriere (2005) 37	EPIDOS	CD	FRA	545	79 (76–81)	100	7 y	—	—	LB	≥1 loss	
Cesari (2015) 38	InCHIANTI	CD	ITA	991	73.9 ± 6.7	57.0	9 y	Katz	≥1 loss	LB	Cont	
Chan (2014) 39	ISCOPE	CD	USA	764	83 (79–87)	68.2	1 y	GARS	Cont	GARS	Cont	
Chaudhry (2010) 40	CHS	CD	USA	5888	72.4^a^	57.6	7 y	Own Q	≥1 loss	—	—	
Chu (2006) 41	—	CD	HKG	1419	73.1 ± 6.2	49.5	1 y	M BI	≥1 loss	LB	≥1 loss	
Cooper (2011) 42	LASA	CD	NLD	1532	70.0 ± 8.5	54.8	3 y	Own Q	≥1 loss	—	—	
Corsonello (2012) 43	PVC	IP	ITA	506	80.1 ± 5.9	54.3	1 y	M Katz	≥1 loss	—	—	
Costanzo (2018) 44	InCHIANTI	CD	ITA	709	73.4 ± 6.5	56.3	6 y	Katz	≥1 loss	—	—	
Den Ouden (2013) 45	PROFIEL	CD	NLD	625	62.3 ± 8.9	49.0	10 y	M Katz	≥1 loss	—	—	
Denkinger (2010) 46	IRIE	CD	DEU	161	82 (58–93)	72.7	3 w	BI	Cont	—	—	
Di Monaco (2015) 47	—	CD	ITA	193	80.0 ± 7.7	100	6 m	BI	≥15 loss	—	—	
Donoghue (2014) 48	TILDA	CD	IRL	1819	72.8 ± 6.1	52.6	2 y	Own Q	≥1 loss	Own Q	≥1 loss	
Duchowny (2018) 49	HRS	CD	USA	8467	74.6 ± 7.0^a^	57.0	2 y	Own Q	≥1 loss	—	—	
Fantin (2007) 50	—	CD	ITA	159	71.4 ± 2.3^a^	61.0	6 y	Own Q	≥1 loss	Own Q	≥1 loss
Femia (1997) 51	OCTO Project	CD	SWE	95	86.8 ± 2.3	74.0	4 y	Own Q	Cont	Own Q	Cont
Fujiwara (2016) 52	TMIG‐LISA	CD	JPN	981	71.5 ± 5.2	58.1	8 y	Own Q	≥1 loss	—	—
Giampaoli (1999) 53	FINE	CD	ITA	140	76.5 ± 3.4^a^	0	4 y	WHO scale	≥1 loss	WHO scale	≥1 loss
Gill (1996) 54	PS	CD	USA	775	79.1 ± 5.0	74	3 y	M Katz	≥1 loss	—	—
Gill (2009) 55	PEP	CD	USA	722	78.4 ± 5.2	62.4	11 y	Own Q	≥1 loss	—	—
Guralnik (2000) 56	EPESE	CD	USA	2478	—	—	6 y	Own Q	≥1 loss	—	—
Hansen (1999) 57	—	PB	USA	73	80.4 ± 7.0	66.0	1 m	Katz	≥1 loss	M LB	≥1 loss
Heiland (2016) 58	SNAC‐K	CD	SWE	3060	73.7 ± 10.8	63.7	6 y	Own Q	≥1 loss	—	—
Hirani (2015) 59	CHAMP	CD	AUS	1819	77.3 ± 5.8^a^	0	5 y	M Katz	≥1 loss	—	—
Hirani (2017) 60	CHAMP	CD	AUS	1685	76.9 ± 5.5	0	5 y	M Katz	≥1 loss	LB	≥1 loss
Hoeymans (1996) 61	Zitphen Elderly	CD	NLD	303	75.8 ± 5.4	0	3 y	M WHO	≥1 loss	M WHO	≥1 loss
Hong (2016) 62	—	CD	KOR	8000	72.5 ± 5.5	59.4	3 y	—	—	KIADL	≥1 loss
Idland (2013) 63	—	CD	NOR	113	79.4 ± 2.9	100	9 y	M A PADL‐H scale	≥1 loss	—	—
Ishizaki (2000) 64	LISA	CD	JPN	583	70.9 ± 4.9	55.9	3 y	Own Q	≥1 loss	TMIG IC	≥1 loss
Janssen (2006) 65	CHS	CD	USA	3694	73.5^a^	53.2	8 y	Own Q	≥1 loss	—	—
Jonkman (2018) 16	InCHIANTI, LASA	CD	ITA, NLD	798	67.5 ± 2.1^a^	53.8	9 y	Own Q	≥1 loss	Own Q	≥1 loss
Kempen (1998) 66	GLAS	CD	NLD	557	72.4 ± 7.7^a^	74.7	2 y	GARS	Cont	—	—
Kozicka (2016) 67	—	CD	POL	41	69.8 ± 9.0	41.5	1 y	Katz	Cont	LB	Cont
Kwon (2012) 68	—	IP	USA	204	71.1 ± 5.3	57.8	1 y	HAQ	≥1 loss	—	—
Legrand (2014) 69	BFc80+	CD	BEL	431	84.4 ± 3.5	63.0	34 m	Own Q	≥3 loss	—	—
Lopez‐Teros (2014) 70	Coyoacan	CD	MEX	133	75.5 ± 4.7	53.4	1 y	Own Q	≥1 loss	Own Q	≥1 loss
McGrath (2018) 71	HEPESE	CD	USA	672	81.7 ± 4.1	64.6	2 y	M Katz	≥1 loss	OARS and RB	≥1 loss
Minneci (2015) 72	ICARe Dicomano	PB, HF	ITA	561	72.9 ± 7.1^a^	57.6	3 y	Own Q	≥1 loss	—	—
Moen (2018) 73	—	PD	NOR	115	86.0 ± 5.9	55.0	3 w	Nor BI	Cont	—	—
Onder (2005) 74	WHAS	CD	USA	884	78.7 ± 8.0	100	3 y	Own Q	≥1 loss	—	—
Ostir (1998) 75	EPESE	CD	USA	1342	73.3	53.0	2 y	Own Q	≥1 loss	—	—
Peel (2014) 76	—	TCP	AUS	351	79.0 ± 8.8	65.8	6 m	interRAC HC	≥1 loss	—	—
Pisters (2012) 77	—	IP	NLD	216	66.1 ± 8.5	72.2	5 y	WOMAC	Cont	—	—
Purser (2005) 78	VA	IP	USA	1388	74 ± 6.0	2.0	1 y	Katz	Cont	LB	Cont
Rajan (2012) 79	CNDS	CD	USA	5317	73.2 ± 6.4	61.0	8 y	Katz	≥1 loss	—	—
Rantanen (1999) 80	HPP, HAAS	CD	USA	6089	54.0 ± 5.5	0	25 y	Own Q	≥1 loss	—	—
Rantanen (2002) 81	NORA75	CD	DNK, SWE, FIN	567	75+, NR	60.0	5 y	Own Q	≥1 loss	—	—
Rodriguez‐Pascual (2017) 82	—	PD, HF	ESP	497	85.2 ± 7.3	61.0	1 y	Katz	≥1 loss	—	—
Rothman (2008) 83	PEP	CD	USA	754	78.4 ± 5.3	64.6	8 y	Own Q	≥1 loss	—	—
Sakamoto (2016) 84	TLAS	CD	JPN	188	80.2 ± 3.9^a^	65.4	2 y	Own Q	≥1 loss	—	—
Sanchez‐Martinez (2016) 85	Penagrade cohort	CD	ESP	607	77.0 ± 7.6	50.9	4 y	Own Q	≥1 loss	—	—
Sanchez‐Rodrigeuz (2014) 86	—	IP	ESP	99	84.6 ± 6.6	61.6	3 m	BI	Cont	—	—
Sarkisian (2000) 87	SOF	CD	USA	6632	73.0 ± 4.9	100	4 y	Own Q	≥1 loss	Own Q	≥1 loss
Sarkisian (2001) 88	SOF	CD	USA	89	72.4 ± 4.5^a^	100	4 y	NHIS	≥1 loss	—	—
Schoenenberg (2013) 89	—	IP, TAVI	CHE	119	83.4 ± 4.6	55.5	6 m	Katz	≥1 loss	LB	≥1 loss
Seidel (2011) 90	SHARE	CD	EU	6841	72 ± 6.0	52.5	2 y	—	—	Own Q	≥1 loss
Shimada (2010) 91	E‐SAS project	CD	JPN	436	79.2 ± 6.8	72.5	1 y	—	—	TMIG IC	≥1 loss
Shimada (2015) 92	OSHPE	CD	JPN	4081	71.7 ± 5.3	51.6	2 y	LTIC	≥1 loss	—	—
Shinkai (2000) 93	TMIG‐LISA	CD	JPN	748	NR	NR	6 y	Own Q	≥1 loss	—	—
Shinkai (2003) 94	TMIG‐LISA	CD	JPN	601	73.0 ± 5.3	65	4 y	Own Q	≥1 loss	TMIG IC	≥1 loss
Sourdet (2012) 95	REAL.FR	CD, AD	FRA	632	77.8 ± 7.0^a^	72.2	2 y	M Katz	≥0.5 loss	—	—
Stenholm (2014) 96	InCHIANTI	CD	ITA	724	67.1 ± 15.0^a^	54.3	9 y	Own Q	≥1 loss	—	—
Taekema (2010) 18	Leiden 85‐plus	CD	NLD	555	NR	65.0	NR	GARS	≥1 loss	GARS	≥1 loss
Takuhiro (2017) 97	Hizen‐Oshima	CD	JPN	104	69.3 ± 3.0	100	9 y	Composite	≥3 loss	—	—
Tanimoto (2013) 98	—	CD	JPN	716	73.2 ± 6.1^a^	65.8	2 y	M Katz	≥1 loss	—	—
Terhorst (2017) 99	—	OP	USA	256	78.9 ± 5.1	100	6 m	PASS	≥1 loss	PASS	≥1 loss
Tinetti (2005) 100	PEP, PS	CD	USA	1471	78.8 ± 5.2^a^	70.8	3 y	—	—	LB	≥1 loss
Volpato (2011) 101	—	IP	ITA	87	77.4 ± 6.5^a^	49.0	3 m	Own Q	Cont	M LB	Cont
Wennie Huang (2010) 102	—	CD	USA	110	80.3 ± 7.0	70.9	18 m	NHIS	≥1 loss	—	—
Zhang (2013) 103	InCHIANTI	CD	ITA	562	71.4 ± 5.7	47.9	3 y	Own Q	≥1 loss	Own Q	≥1 loss
Zoico (2007) 104	—	CD	ITA	145	71.7 ± 2.3^a^	58.6	2 y	Composite	≥1 loss	—	—

*Cohort*: 3C, Three City Study; BFc80+, BELFRAIL; Boston RISE, Boston Rehabilitative Impairment Study of the Elderly; CHAMP, The Concord Health and Ageing in Men Project; CHS, Cardiovascular Health Study; CNDS, Chicago Neighbourhood and Disability Study; Coyoacan, Mexica Study of Nutritional and Psychosocial Markers of Frailty among Community‐dwelling Elderly; EPESE, Established Populations for the Epidemiological Study for the Elderly; EPIDOS, epidemiology of osteoporosis; E‐SAS project, Elderly Status Assessment Set; FINE, Finland, Italy, Netherlands Elderly; GLAS, Groningen Longitudinal Ageing Study; HAAS, Honolulu Asia Aging Study; HEPESE, Hispanic Established Populations for the Epidemiological Study for the Elderly; HPP, Honolulu Heart Program; HRS, Health and Retirement Study; ICARe Dicomano, Insufficienza Cardiaca negi Anziani Residenti a Dicomano; InCHIANTI, Invecchiare in Chianti; ISCOPE, Integrated Systematic Care for Older People; LASA, Longitudinal Aging Study Amsterdam; NMAPS, New Mexico Aging Process Study; NORA75, Nordic Research on Aging 75 study; OSHPE, Obu Study of Health Promotion for the Elderly; PEP, Precipitating Events Project; PS, Project Safety; PVC, PharmacosurVeillance in the elderly Care; REAL.FR, Reseau sur la maladie Alzheimer Francais; SABE, Saude, Bem‐Estar e Envelhecimento; SITE, Sources of Independence in the Elderly; SHARE, The Survey of Health, Ageing and Retirement in Europe; SNAC‐K, Swedish National study on Aging and Care in Kungsholemn; SOF, Study of Osteoporotic Fractures; TILDA, The Irish Longitudinal Study of Ageing; LISA, Longitudinal Interdisciplinary Study on Aging; TLAS, Tosa Longitudinal Aging Study; TMIG‐LISA, Tokyo Metropolitan Institute of Gerontology Longitudinal Interdisciplinary Study on Ageing; VA, Department of Veterans Affairs; WHAS, Women's Health and Aging Study. *Setting*: AD, Alzheimer's disease; CD, community‐dwelling; OP, outpatients; IP, inpatients; TAVI, transcatheter aortic valve implantation; TCP, transitional care program; PB, population based; PD, post‐discharge; HF, heart failure. *Country*: AUS, Australia; BEL, Belgium; BRA, Brazil; CHE, Switzerland; DEU, Germany; DNK, Denmark; ESP, Spain; EU, Europe; FIN, Finland; FRA, France; HKG, Hong Kong; IRL, Ireland; ISR, Israel; ITA, Italy; JPN, Japan; KOR, Korea; MEX, Mexico; NLD, Netherlands; NOR, Norway; POL, Poland; SWE, Sweden; USA, United States. *Age*: presented as mean ± SD or median (range) or (IQR); range, percentage; —, not applicable or reported; F, female; FU, follow‐up duration; D, day(s); M, month(s); Y, year(s). *ADL*: —, not applicable or reported; BI, Barthel Index; Cont, continuous; GARS, Groningen Activities Restriction Scale; HAQ, Stanford Health Assessment Questionnaire; interRAC HC, interRAC Home Care; Nor BI, Norwegian Barthel Index; M A PADL‐H scale, modified Avlund Physical ADL‐H scale; M Katz, modified Katz Index; M BI, modified Barthel Index; M WHO, modified World Health Organization scale; LLFDI‐FC, Functional Component of the Late‐Life Function and Disability Instrument; LTCI, long‐term care insurance system; PASS, Performance Assessment of Self‐Care Skills; WHO, World Health Organization; WOMAC, Western Ontario and McMaster Universities Osteoarthritis Index. *IADL*: —, not applicable or reported; AMPS, assessment of motor and process skills; GARS, Groningen Activities Restriction Scale; KIADL, Korean IADL; LB, Lawton and Brody; M LB, modified Lawton and Brody; M WHO, modified WHO scale; NHIS, National Health Interview Survey; OARS, Older Americans Resources and Services; RB, Rosow‐Breslau scale; TMIG‐IC, Tokyo Metropolitan Institute of Gerontology Index of Competence.

a Calculated mean ± SD or mean from information provided.

### Qualitative analysis

Muscle mass was reported in 13 articles,^28^, ^32^, ^35^, ^36^, ^37^, ^38^, ^50^, ^59^, ^60^, ^65^, ^86^, ^98^, ^104^ six using dual‐energy X‐ray absorptiometry, five using bioelectrical impedance analysis, one using computed tomography, and one did not report the measurement method (*Table* 2). Low muscle mass was positively associated with worsening ADL in 5/9 articles and IADL in 5/5 at follow‐up.

**Table 2:** Muscle mass as predictor of activities of daily living or instrumental activities of daily living

First author (year) [ref]	*N*	Tool	Measure	Units	Cut‐offs	AM	MA
Amigues (2013) 28	975	DXA	SMI	kg/m^2^	SD (NR)	A	Y
					Quartile^a^	A	N
	975	DXA	LM	kg	SD (NR)	A	N
Baumgartner (2004) 32	451	DXA	SMI	kg/m^2^	M: 7.26. F: 5.45	A	N
Bianchi (2015) 35	538	BIA	SMI	kg/m^2^	M: 8.87. F: 6.42	A	Y
Broadwin (2001) 36	1051	BIA	FFM	%	Quintile^b^	A	N
Carriere (2005) 37	545	—	LM/BM	—	<0.54, 0.54–0.63, ≥0.63	A	N
Cesari (2015) 38	991	CT	Muscle density	mg/cm^3^	SD (M: 3.32. F: 3.60)	A	N
Fantin (2007) 50	159	DXA	FFM	kg	Continuous	U	N
Hirani (2015) 59	1819	DXA	ALM	kg	<19.75	A	N
Hirani (2017) 60	1685	DXA	ALM/BMI	—	<0.789	A	Y
Janssen (2006) 65	3694	BIA	MM	kg	Quartile (NR)	A	N
	3694	BIA	SMI	kg/m^2^	M: <10.75. F: <6.75	A	Y
Sanchez‐Rodrigeuz (2014) 86	99	BIA	FFM	kg	Continuous	A	N
	99	BIA	LBM	kg	Continuous	A	N
Tanimoto (2013) 98	716	BIA	AMI	kg/m^2^	M: <7.0. F: <5.8	U	Y
Zoico (2007) 104	145	DXA	AMI	kg/m^2^	<7.6	A	N

*Measure*: —, not applicable or reported; ALM, appendicular lean mass; AMI, appendicular mass index; BIA, bioelectrical impedance analysis; BM, body mass; CT, computed tomography; DXA, dual‐energy X‐ray absorptiometry; FFM, fat free mass; LBM, lean body mass; LM, lean mass; MM, muscle mass; SMI, skeletal muscle index. *Cut‐off*: NR, not reported; SD, standard deviation. Expressed as either dichotomous, ranges for specific tertiles, quartiles, quintiles, or categories or per unit or score. AM, adjustment model denotes whether the model in the meta‐analysis was: A, adjusted; or U, unadjusted. MA, meta‐analysis; N, no; Y, yes.

a ≥6.72, 6.30–6.72, 5.82–6.30, <5.82.

b M: 35.5–75.6, 75.7–78.0, 78.1–80.2, 80.3–82.8, 82.9–93.0. F: 45.6–67.0, 67.1–69.4, 69.5–71.8, 71.9–74.7, 74.8–88.0.

**Table 3:** Muscle strength as predictor of activities of daily living or instrumental activities of daily living

First author (year) [ref]	*N*	Measure	Units	Cut‐offs	AM	MA
Abete (2017) 24	907	HGS	NR	Dich (NR)	A	Y
	907	SS	NR	Dich (NR)	A	N
Al snih (2004) 25	2493	HGS	kg	Continuous	U	Y
				Quartile^a^	U	Y
Alexandre (2012) 27	1634	HGS	kg	Continuous	A	Y
Amigues (2013) 28	975	HGS	kPa	SD (NR)	A	N
Beloosesky (2009) 34	93	HGS	kg	Continuous	U	N
Bianchi (2015) 35	538	HGS	kg	BMI specific^b^	A	N
Carriere (2005) 37	545	HGS	kPa	<47	A	Y
	545	QS	N/cm	<3.52, 3.52–4.95, ≥4.95	A	N
Cesari (2015) 38	991	HGS	kg	SD (M: 10.11. F: 7.49)	A	N
	991	AE	kg	SD (M: 9.79. F: 8.35)	A	N
Chan (2014) 39	570	HGS	kg	Continuous	A	N
	570	QS	kg	Continuous	A	N
Chaudhry (2010) 40	5888	HGS	kg	Lowest quintile for sex and BMI (NR)	A	Y
Costanzo (2018) 44	709	HGS	—	Lowest quintile for sex and BMI (NR)	U	Y
Den Ouden (2013) 45	625	HGS	kg	Per 10	A	N
	625	QS	Nm	Per 10	A	N
Di Monaco (2015) 47	193	HGS	kg	SD (5.7)	A	N
Duchowny (2018) 49	8467	HGS	kg	WM: <35, BM: <40, WW: <22, BW: <31	A	Y
Femia (1997) 51	95	HGS	kPa	Continuous	U	N
Giampaoli (1999) 53	140	HGS	kPa	Continuous	A	N
Gill (2009) 55	722	HGS	kg	BMI specific^c^	A	Y
Hirani (2015) 59	1819	HGS	kg	<26	A	N
Ishizaki (2000) 64	468	HGS	kg	Continuous	A	Y
Kozicka (2016) 67	41	HGS	kg	Continuous	U	N
Kwon (2012) 68	204	HGS	kg	BMI specific^d^	A	N
Legrand (2014) 69	309	HGS	kg	Tertile^e^	A	Y
Lopez‐Teros (2014) 70	133	HGS	kg	Continuous	A	N
McGrath (2018) 71	672	HGS	kg	Continuous	A	N
Minneci (2015) 72	453	HGS	kg	Continuous	A	Y
Moen (2018) 73	115	HGS	kg	Continuous	A	N
Onder (2005) 74	458	HGS	kg	SD (5.9)	A	N
Pisters (2012) 77	216	KE	N/kg	SD (0.6)	A	N
Rantanen (1999) 80	6089	HGS	kg	Tertile^f^	A	N
Rantanen (2002) 81	553	HGS	N	M: <392. F: <225	U	Y
	554	AF	N	M: <274, <348. F: <159, <198	A	N
	550	KE	N	M: <363, <449. F: <225, <287	A	N
	546	TE	N	M: <542, <631. F: <271, <393	A	N
	538	TF	N	M: <472, <571. F: <231, <330	A	N
Rodriguez‐Pascual (2017) 82	277	HGS	kg	Lowest quintile for sex and BMI (NR)	A	Y
Rothman (2008) 83	754	HGS	kg	BMI specific^g^	A	Y
Sanchez‐Rodrigeuz (2014) 86	99	HGS	kg	Continuous	U	N
Sarkisian (2000) 87	6632	HGS	kg	Lowest Quintile (NR)	A	Y
Sarkisian (2001) 88	89	HGS	kg	Decile (NR)	A	N
Seidel (2011) 90	6670	HGS	kg	<26	U	Y
Shinkai (2000) 93	513	HGS	kg	Age specific^h^	U	Y
Shinkai (2003) 94	601	HGS	kg	Quartile decrease	A	Y
Taekema (2010) 18	555	HGS	kg	Continuous	A	N
Tanimoto (2013) 98	716	HGS	kg	Lowest quartile (NR)	U	N
Wennie Huang (2010) 102	65	HGS	kg	Continuous	A	Y

*Measure*: —: not applicable or reported; AE, ankle extension; AF, arm flexion; BMI, body mass index; HGS, handgrip strength; KE, knee extension; QS, quadriceps strength; SS, shoulder strength; TE, trunk extension; TF, trunk flexion. *Cut‐offs*: NR, not reported; SD, standard deviation. Expressed as either dichotomous, ranges for specific tertiles, quartiles, quintiles, or categories or per unit or score. AM, adjustment model denotes whether the model in the meta‐analysis was: A, adjusted; or U, unadjusted. MA, meta‐analysis; N, no; Y, yes.

a M: <22.00, 21.01–30.00, 30.01–35.00, ≥35.01. F: <14.00, 14.01–18.20, 18.21–22.50, ≥22.51.

b M: ≤24: ≤29, 24.1–28: ≤30, >30: ≤32. F: ≤23: ≤17, 23.1–26: ≤17.3, 26.1–29: ≤21.

c M: ≤24: ≤29, 24.1–26: ≤30, 26.1–28: ≤30, >28: ≤32. F: ≤23: ≤17, 23.1–26: ≤17.3, 26.1–29: ≤18, >29: ≤21.

d M: <25, 25.0–29.9, 30.0–39.9, >40. F: <15, 15.0–19.9, 20.0–24.9, >25.

e M: <25.3, 25.4–33.2, >33.3. F: <15.0, 15.1–20.0, >20.1.

f <37.0, 37.0–42.0, >42.0.

g M: ≤24: ≤29, 24.1–26: ≤30, 26.1–28: ≤30, >28: ≤32. F: ≤23: ≤17, 23.1–26: ≤17.3, 26.1–29: ≤18, >29: ≤21.

h M: 65–74: <37, ≥75: <30. F: 65–74: <22, ≥75: <20.

Muscle strength was investigated in 41^18^, ^24^, ^25^, ^27^, ^28^, ^34^, ^35^, ^37^, ^38^, ^39^, ^40^, ^44^, ^45^, ^47^, ^49^, ^51^, ^53^, ^55^, ^59^, ^64^, ^67^, ^68^, ^69^, ^70^, ^71^, ^72^, ^73^, ^74^, ^77^, ^80^, ^81^, ^82^, ^83^, ^86^, ^87^, ^88^, ^90^, ^93^, ^94^, ^98^, ^102^ articles as a predictor of ADL and IADL (*Table* 3). Handgrip strength was used as a measure of muscle strength in 40 articles, five using quadriceps or knee strength and each of the following were used in single articles: shoulder strength, ankle and torso flexion, and extension. Low muscle strength was positively associated with worsening ADL in 22/34 and IADL in 8/9 at follow‐up.

A total of 62 articles^16^, ^26^, ^27^, ^28^, ^29^, ^30^, ^31^, ^33^, ^35^, ^37^, ^38^, ^40^, ^41^, ^42^, ^43^, ^44^, ^45^, ^46^, ^48^, ^49^, ^52^, ^54^, ^55^, ^56^, ^57^, ^58^, ^59^, ^61^, ^62^, ^63^, ^66^, ^68^, ^69^, ^70^, ^71^, ^72^, ^73^, ^74^, ^75^, ^76^, ^78^, ^79^, ^82^, ^83^, ^84^, ^85^, ^87^, ^89^, ^90^, ^91^, ^92^, ^93^, ^94^, ^95^, ^96^, ^97^, ^98^, ^99^, ^100^, ^101^, ^102^, ^103^ reported physical performance as a predictor of ADL and IADL (*Table* 4). Gait speed was the most reported measure, with a total of 37 articles, followed by the composite test short physical performance battery (SPPB), which was reported in 14 articles. Other measures of physical performance included a variety of balance (*n* = 15), chair stand (*n* = 10), timed up and go (*n* = 8), and functional reach tests (*n* = 2) as well as tests that involve multiple assessments (*n* = 3). Poor physical performance was positively associated with worsening ADL in 37/49 articles and IADL in 9/11 at follow‐up: SPPB (10/13, 2/2), gait speed (27/33, 7/8), one leg balance (3/4, 0/0), and chair stand test (4/9, 0/1).

**Table 4:** Physical performance as predictor of activities of daily living or instrumental activities of daily living

First author (year) [ref]	*N*	Measure	Units	Cut‐offs	AM	MA
Albert (2015) 26	347	Gait	m/s	Quartile^a^	A	N
Alexandre (2012) 27	1634	OLB	s	Continuous	A	N
	1634	CST	s	Continuous	A	N
Amigues (2013) 28	975	Gait	m/s	SD (0.22)	A	N
	974	Balance	s	Tertile^b^	A	N
Arnau (2016) 29	252	SPPB	points	<7	U	Y
Artaud (2015) 30	3814	Fast Gait	m/s	SD (0.22)	A	N
	3814	Change in FG	m/s	SD (0.013)	A	N
Basic (2017) 31	1693	TUG	s	Continuous	U	N
Beauchamp (2015) 33	430	SPPB	points	Continuous	U	N
	430	Gait	m/s	Continuous (0.1)	U	N
	428	400 m walk	min	Continuous	U	N
	413	Stair climb	watts	Continuous	U	N
Bianchi (2015) 35	538	Gait	m/s	<0.8	A	N
Carriere (2005) 37	545	Gait	m/s	<0.78	A	Y
	545	Standing balance	—	Tertile^c^	A	N
	545	Dynamic balance	—	Cat (3)	A	N
	545	CST	s	<13 s	A	Y
	545	Foot tapping	—	Tertile	A	N
Cesari (2015) 38	991	Gait	m/s	SD (M: 0.24. F: 0.23)	A	Y
Chaudhry (2010) 40	5888	Gait	m/s	Lowest quintile for sex and height (NR)	A	Y
Chu (2006) 41	1338	Gait	m/s	<0.65 m/s	A	Y
Cooper (2011) 42	1425	Timed walk test	—	Continuous	A	N
	1425	Tandem stand	—		A	N
	1425	Cardigan test	s		A	N
	1425	CST	s		A	N
Corsonello (2012) 43	506	SPPB	points	Continuous	A	Y
				<9	U	Y
Costanzo (2018) 44	709	Gait	m/s	Lowest quintile for sex and height (NR)	U	Y
	709	Balance	s	Lowest quintile for sex and height (NR)	U	N
Den Ouden (2013) 45	625	SPPB	points	Continuous	A	Y
Denkinger (2010) 46	161	Change in gait	m/s	Continuous	A	N
Donoghue (2014) 48	1391	Gait	m/s	Continuous (0.1)	U	N
	1391	TUG	s	Continuous	U	N
Duchowny (2018) 49	8467	Gait	m/s	<0.8	A	Y
Fujiwara (2016) 52	981	Gait	m/s	Tertile (NR)	A	Y
Gill (1996) 54	775	Own test	—	Quartile (25%)	U	N
Gill (2009) 55	722	SPPB	points	Continuous	A	Y
	722	RGT	s	≤10	U	N
	722	Gait and balance	points	Continuous	U	N
	722	CST	s	Dich (NR)	U	Y
	722	Chair	—	Dich	U	N
	722	Manual dexterity	s	Quartile^d^	U	N
	722	GMC	s	Quartile^e^	U	N
Guralnik (2000) 56	2542	SPPB	points	<10	U	Y
Hansen (1999) 57	73	TUG	s	Tertile^f^	U	N
	73	Tinetti balance	points	Tertile^g^	U	N
Heiland (2016) 58	1971	Gait	m/s	<0.8	A	Y
	1971	OLB	s	<5	A	Y
Hirani (2015) 59	1819	Gait	m/s	≤0.8	A	N
Hoeymans (1996) 61	303	Gait	m/s	<0.73	U	N
	303	CST	s	≤17.2	U	N
Hong (2016) 62	8000	Gait	m/s	<0.6	A	Y
Idland (2013) 63	113	Gait	m/s	Cont (1)	A	Y
	113	FRT	cm	Cont (1)	A	N
	113	Step climb test	cm	Per 10	A	N
Jonkman (2018) 16	798	Gait	m/s	Dich (NR)	A	N
	798	Tandem stand	s	<10 s	A	N
Kempen (1998) 66	557	Walk turn walk	s	Cont	A	N
	557	CST	s	Cont	A	N
	557	CST	s	Cont	A	N
557	Jacket	s	Cont	A	N
Kwon (2012) 68	204	Gait	m/s	Quartile^h^	A	N
	204	Balance	s	FT10, FT1‐9, ST10, StS10	A	N
	204	CST	s	Quartile^i^	A	N
Legrand (2014) 69	308	SPPB	points	M: <10. F: <8	U	Y
Lopez‐Teros (2014) [54]	133	Gait	m/s	Continuous (1)	A	Y
McGrath (2018) 71	672	Gait	m/s	Lowest quintile for sex and height (NR)	A	Y
Minneci (2015) 72	453	Gait	m/s	Continuous (1)	A	Y
	453	SPPB	points	Continuous	A	Y
	453	6MWT	m	Continuous	A	N
Moen (2018) 73	75	TUG	s	Continuous	A	N
Onder (2005) 74	458	Balance	s	SD (10.2)	A	N
	458	CST	s	SD (8.4)	A	N
	458	Gait	m/s	SD (0.31)	A	Y
	458	LE comp	—	SD (0.69)	A	N
	458	Blouse	s	SD (72)	A	N
	458	Purdue	s	SD (10.5)	A	N
	458	UE comp	—	SD (0.49)	A	N
Ostir (1998) 75	1342	SPPB	points	<9	U	Y
	1328	Gait	m/s	<0.8	U	Y
	1342	CST	s	<10.9	U	Y
	1006	Balance	s	FT10, FT2‐10, StS10	U	N
Peel (2014) 76	280	Gait	m/s	Continuous (0.1)	A	Y
Purser (2005) 78	1388	Gait	m/s	Continuous (0.1)	A	N
	1388	Change in gait	m/s	Continuous (0.1)	A	N
Rajan (2012) 79	5317	M SPPB	points	Continuous	A	Y
Rodriguez‐Pascual (2017) 82	218	Gait	m/s	Lowest quintile for sex (NR)	A	Y
Rothman (2008) 83		Gait	m/s	<0.3	A	N
Sakamoto (2016) 84	188	TUG	s	<15 s	A	Y
	188	FRT	cm	<20	A	N
Sanchez‐Martinez (2016) 85	607	SPPB	points	<8	A	N
	607	Gait	m/s	<0.8	A	N
Sarkisian (2000) 87	6632	Gait	m/s	Lowest quintile (NR)	A	N
Schoenenberg (2013) 89	119	TUG	s	<20 s	U	Y
Seidel (2011) 90	1804	Gait	m/s	<0.4	U	Y
Shimada (2010) 91	436	TUG	s	<12	A	Y
Shimada (2015) 92	4081	Gait	—	<1.0	A	N
Shinkai (2000) 93	513	UWS	m/s	Tertile^j^	U	Y
	513	MWS	m/s	Age and sex specific quartiles^k^	A	N
	513	OLB	s	Tertile^l^	U	Y
Shinkai (2003) 94	601	Gait	m/s	Quartile lower	A	N
	601	Fast gait	m/s	Quartile lower	A	N
	601	OLB	s	Quartile lower	A	N
Sourdet (2012) 95	583	OLB	s	<5	A	N
Stenholm (2014) 96	727	Gait	m/s	Continuous (0.1)	A	Y
	727	SPPB	points	Continuous	U	Y
Takuhiro (2017) 97	104	RWS	m/s	SD (0.24)	A	Y
Tanimoto (2013) 98	716	Gait	m/s	Lowest quartile (NR)	U	N
Terhorst (2017) 99	256	Balance	—	NR	U	N
	256	Forward reach	—	NR	U	N
Tinetti (2005) 100	1042	CST	s	Tertile^m^	U	N
Volpato (2011) 101	74	SPPB	points	<8	U	Y
Wennie Huang (2010) 102	65	SPPB	points	Continuous	A	Y
	65	Gait	m/s	Continuous (1)	A	Y
	65	BBS	points	Continuous	A	N
	65	TUG	s	Continuous	A	N
Zhang (2013) 103	504	CST	s	<11.2	A	Y

*Physical Performance*: —, not applicable or reported; 6MWT, 6 min walk test; BBS, Berg Balance Scale; CST, chair stand test; OLB, one leg balance; FRT, functional reach test; FT, full tandem; MWS, maximum walking speed; POMA, performance oriented mobility assessment; PPT, physical performance test; RGT, rapid gait test; RWS, regular walking speed; SPPB, short physical performance battery; ST, semi tandem; StS, side to side; TUG, timed up and go; UWS, usual walking speed; WS, walking speed. *Cut‐offs*: NR, not reported; SD, standard deviation. Expressed as either dichotomous, ranges for specific tertiles, quartiles, quintiles, or categories or per unit or score unless otherwise stated in brackets. AM, adjustment model denotes whether the model in the meta‐analysis was: A, adjusted; or U, unadjusted. MA, meta‐analysis; N, no; Y, yes.

a ≥1, 0.74–0.99, 0.57–0.73, <0.57.

b <2, 3–9, ≥10.

c Full tandem, semi tandem, side to side.

d <21.8, 21.8–24.3, 24.4–27.5, ≥27.6.

e <8.8, 8.8–10.3, 10.4–12.4, ≥12.5.

f <20, 20–40, ≥40.

g 15–27, 28–38, 39–41.

h M: >4.5, 4.0–4.5, 3.0–3.9, <3. F: >5, 4.0–5.0, 3.0–3.9, <3.

i M: >20, 17.0–19.0, 11–16.9. F: >21, 18.0–20.9, 12.0–17.9, <12.

j M: ≤1.08, ≥75: ≥0.82. F: ≤0.9, ≥75: ≤0.69.

k M: 65–74: ≤1.81, 1.82–2.10, 2.11–2.36, ≥2.37. ≥75: ≤1.34, 1.35–1.64, 1.65–1.99, ≥2.00. F: 65–75: ≤1.45, 1.46–1.70, 1.71–1.98, ≥1.97. ≥75: ≤1.08, 1.09–1.34, 1.35–1.62, ≤1.63.

l M: ≤18, ≥75: ≤5. F: ≤7, ≥75: ≤16.

m <9, 9–14, >14.

### Quality assessment

A complete breakdown of the NOS can be found in *Table* 5. The majority of the articles were of high quality (46/83).

**Table 5:** Quality assessment of included studies using a modified Newcastle–Ottawa Scale (NOS)

First author (year) [ref]	Selection			Comp	Outcome			Total score
	Q1	Q2^a^	Q3	Q1	Q1	Q2	Q3	
Abete (2017) 24	1		1	1	1	1	1	6/7
Al snih (2004) 25	1		1	2	1	1	1	7/7
Albert (2015) 26	1		1	0	1	1	1	5/7
Alexandre (2012) 27	1		1	0	1	1	1	5/7
Amigues (2013) 28	1	1	1	2	1	1	1	8/8
Arnau (2016) 29	1		1	2	1	1	1	7/7
Artaud (2015) 30	1		1	2	1	1	1	7/7
Basic (2017) 31	1		1	0	1	0	0	3/7
Baumgartner (2004) 32	1	1	1	2	1	1	1	8/8
Beauchamp (2015) 33	1		1	0	1	1	0	4/7
Beloosesky (2009) 34	0		1	0	1	1	1	4/7
Bianchi (2015) 35	1	1	1	2	1	1	1	8/8
Broadwin (2001) 36	1		1	2	1	1	1	7/7
Carriere (2005) 37	1		1	2	1	1	1	7/7
Cesari (2015) 38	1	1	1	2	1	1	1	8/8
Chan (2014) 39	1		1	1	1	1	1	6/7
Chaudhry (2010) 40	1		1	2	1	1	1	7/7
Chu (2006) 41	1		1	2	1	1	1	7/7
Cooper (2011) 42	1		1	2	1	1	1	7/7
Corsonello (2012) 43	0		1	2	1	1	1	6/7
Costanzo (2018) 44	1		1	0	1	1	1	5/7
Den Ouden (2013) 45	1		1	2	1	1	1	7/7
Denkinger (2010) 46	1		1	2	1	0	1	6/7
Di Monaco (2015) 47	1		1	2	1	1	1	7/7
Donoghue (2014) 48	1		1	0	1	1	1	5/7
Duchowny (2018) 49	1		1	2	1	1	1	7/7
Fantin (2007) 50	1		1	0	1	1	1	5/7
Femia (1997) 51	1		1	0	1	1	1	5/7
Fujiwara (2016) 52	1		1	2	1	1	1	7/7
Giampaoli (1999) 53	1		1	2	1	1	1	7/7
Gill (1996) 54	1		1	2	1	1	1	7/7
Gill (2009) 55	1		1	2	1	1	1	7/7
Guralnik (2000) 56	1		1	2	1	1	1	7/7
Hansen (1999) 57	1		1	0	1	0	1	4/7
Heiland (2016) 58	1		1	2	1	1	1	7/7
Hirani (2015) 59	1	1	1	2	1	1	1	8/8
Hirani (2017) 60	1	1	1	2	1	1	1	8/8
Hoeymans (1996) 61	1		1	1	1	1	1	6/7
Hong (2016) 62	1		1	2	1	1	1	7/7
Idland (2013) 63	1		1	2	1	1	1	7/7
Ishizaki (2000) 64	1		1	2	1	1	1	7/7
Janssen (2006) 65	1	1	1	2	1	1	1	8/8
Jonkman (2018) 16	1		1	2	1	1	1	7/7
Kempen (1998) 66	1		1	1	1	1	1	6/7
Kozicka (2016) 67	1		1	0	1	1	0	4/7
Kwon (2012) 68	0		1	2	1	1	1	6/7
Legrand (2014) 69	1		1	2	1	1	1	7/7
Lopez‐Teros (2014) 70	1		1	2	1	1	1	7/7
McGrath (2018) 71	1		1	2	1	1	1	7/7
Minneci (2015) 72	0		1	2	1	1	1	6/7
Moen (2018) 73	1		1	2	1	0	1	6/7
Onder (2005) 74	1		1	1	1	1	1	6/7
Ostir (1998) 75	1		1	2	1	1	0	6/7
Peel (2014) 76	0		1	1	1	1	0	4/7
Pisters (2012) 77	0		1	2	1	1	1	6/7
Purser (2005) 78	0		1	2	1	1	1	6/7
Rajan (2012) 79	1		1	2	1	1	1	7/7
Rantanen (1999) 80	1		1	2	1	1	1	7/7
Rantanen (2002) 81	1		1	1	1	1	1	6/7
Rodriguez‐Pascual (2017) 82	0		1	1	1	1	1	5/7
Rothman (2008) 83	1		1	2	1	1	1	7/7
Sakamoto (2016) 84	1		1	2	1	1	1	7/7
Sanchez‐Martinez (2016) 85	1		1	2	1	1	1	7/7
Sanchez‐Rodrigeuz (2014) 86	0	1	1	0	1	1	1	5/8
Sarkisian (2000) 87	1		1	2	1	1	1	7/7
Sarkisian (2001) 88	1		1	2	1	1	1	7/7
Schoenenberg (2013) 89	0		1	0	1	1	1	4/7
Seidel (2011) 90	1		1	2	1	1	1	7/7
Shimada (2010) 91	1		1	2	1	1	1	7/7
Shimada (2015) 92	1		1	2	1	1	0	6/7
Shinkai (2000) 93	1		1	2	1	1	1	7/7
Shinkai (2003) 94	1		1	2	1	1	1	7/7
Sourdet (2012) 95	0		1	2	1	1	1	6/7
Stenholm (2014) 96	1		1	2	1	1	1	7/7
Taekema (2010) 18	1		1	1	1	1	1	6/7
Takuhiro (2017) 97	1		1	0	1	1	1	5/7
Tanimoto (2013) 98	1	1	1	2	1	1	1	8/8
Terhorst (2017) 99	1		1	0	1	1	1	5/7
Tinetti (2005) 100	1		1	0	1	1	1	5/7
Volpato (2011) 101	0		1	0	1	1	1	4/7
Wennie Huang (2010) 102	1		1	2	1	1	0	6/7
Zhang (2013) 103	1		1	2	1	1	1	7/7
Zoico (2007) 104	1		1	2	1	1	0	6/7

Comp, comparability.

a Only applied to studies that dichotomized sarcopenic cohorts.

### Meta‐analysis

#### Muscle mass

**Figure 2:**
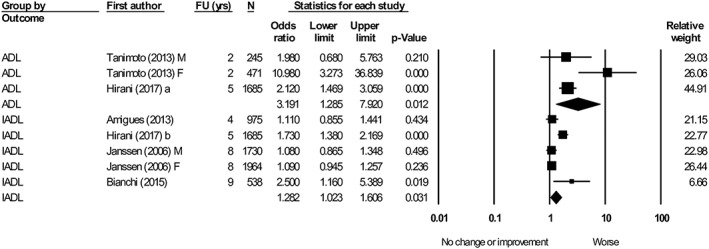
Forest plot showing the association between baseline muscle mass (low vs. high) with activity of daily living (ADL) and instrumental activity of daily living (IADL) at follow‐up. Heterogeneity (*I* ^2^): ADL = 68.8. IADL = 75.8. M, male; F, female. Articles that reported both ADL and IADL were denoted a and b.

Two articles^60^, ^98^ evaluating muscle mass (low vs. high) and its association with ADL were included in the meta‐analysis. Low muscle mass was associated with worsening ADL [OR = 3.19, 95% confidence interval (CI): 1.29–7.92, *I* ^2^ = 68.8]. Four articles^28^, ^35^, ^60^, ^65^ were pooled into a meta‐analysis exploring the effect of muscle mass (low vs. high), which favoured worsening IADL (OR = 1.28, 95% CI: 1.02–1.61, *I* ^2^ = 75.8) (*Figure* 2).

#### Muscle strength

**Figure 3:**
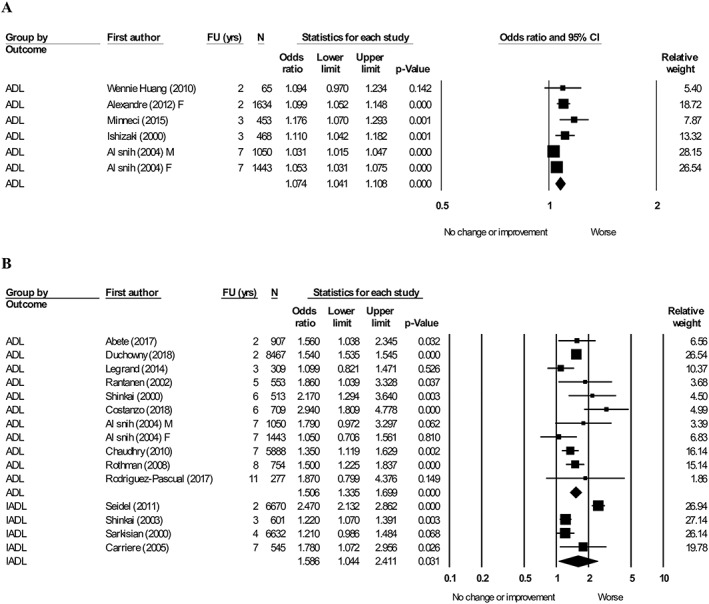
Forest plot showing the association between baseline handgrip strength with activity of daily living (ADL) and instrumental activity of daily living (IADL) at follow‐up. (A) Handgrip strength (per 1 kg lower), heterogeneity (*I* ^2^) = 72.8. (B) Handgrip strength (low vs. high), heterogeneity (*I* ^2^): ADL = 50.0. IADL: 94.7. M, male; F, female.

Six articles^25^, ^27^, ^64^, ^72^, ^102^, ^105^ evaluating the association between handgrip strength (per 1 kg lower) and ADL were pooled, favoured worsening ADL (OR = 1.09, 95% CI: 1.05–1.13, *I* ^2^ = 87.5) (*Figure* 3A). Ten articles^24^, ^25^, ^40^, ^44^, ^49^, ^69^, ^81^, ^82^, ^83^, ^93^ were pooled into meta‐analysis demonstrating the association between low vs. high handgrip strength with ADL (*Figure* 3B). The pooled result again favoured worsening ADL (OR = 1.51, 95% CI: 1.34–1.70, *I* ^2^ = 50.0). Four articles^37^, ^87^, ^90^, ^93^ were pooled measuring handgrip strength (low vs. high) and IADL favouring worsening ADL (OR = 1.59, 95% CI: 1.04–2.41, *I* ^2^ = 94.7) (*Figure* 3B).

#### Physical performance

**Figure 4:**
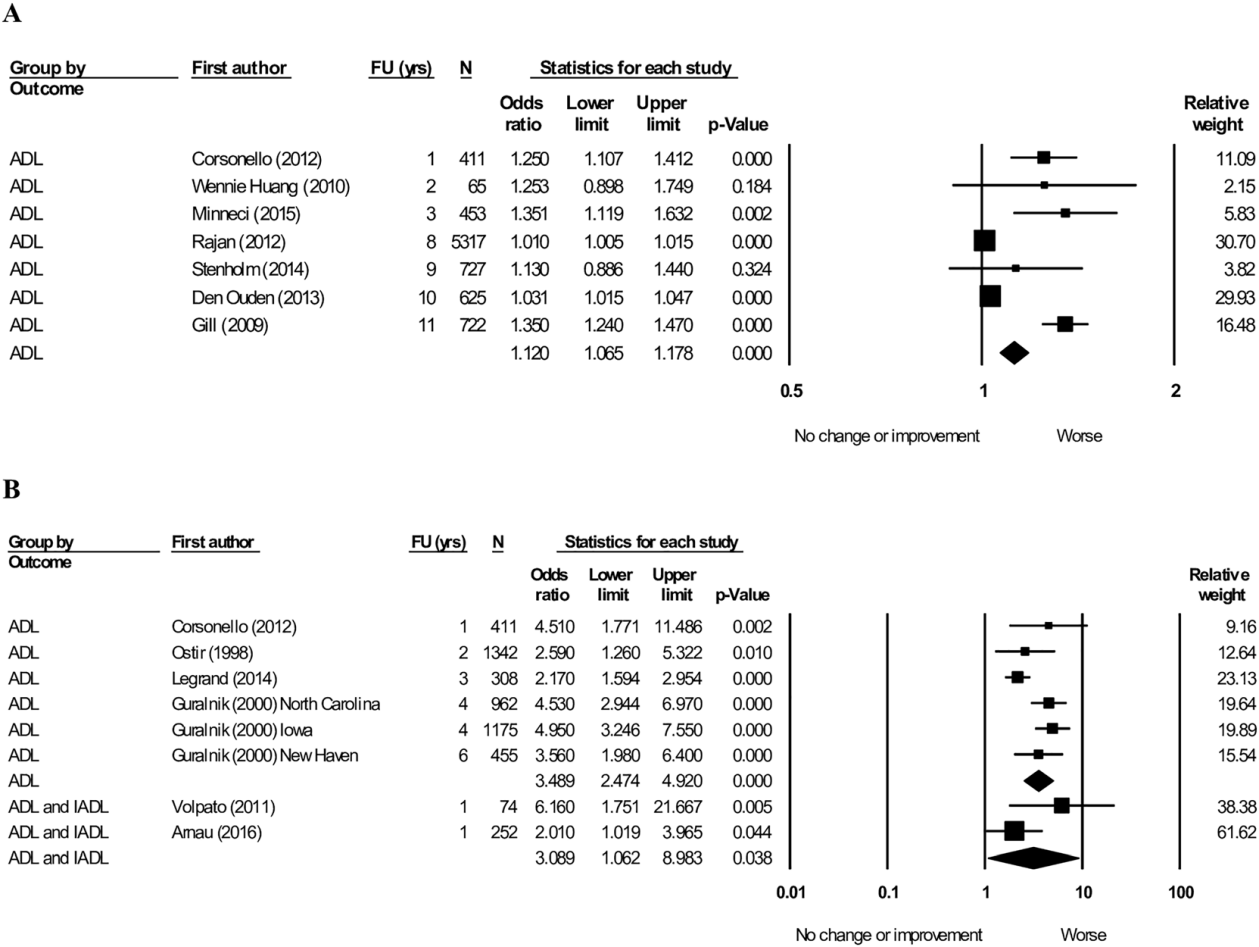
Forest plot showing the association between short physical performance battery (SPPB) with activity of daily living (ADL) and/or instrumental activity of daily living (IADL) at follow‐up. (A) SPPB (per 1 point lower), heterogeneity (*I* ^2^) = 91.8. (B) SPPB (low vs. high), heterogeneity (*I* ^2^): ADL = 63.3. IADL = 57.6.

Seven articles^43^, ^45^, ^55^, ^72^, ^79^, ^96^, ^102^ evaluating the association between SPPB and ADL were pooled, which demonstrated that a lower SPPB score (per one point) is associated with worsening ADL (OR = 1.12, 95% CI: 1.07–1.18, *I* ^2^ = 91.8) (*Figure* 4A). Four articles^43^, ^56^, ^69^, ^75^ were pooled exploring the association between SPPB (low vs. high) and ADL (*Figure* 4B). The pooled effect favoured worsening ADL (OR = 3.49, 95% CI: 2.47–4.92, *I* ^2^ = 63.3). Two articles^29^, ^101^ combined ADL and IADL and showed that SPPB score (low vs. high) favoured a worsening of the combined ADL and IADL measure (OR = 3.09, 95% CI: 1.06–8.98, *I* ^2^ = 57.6) (*Figure* 4B).

**Figure 5:**
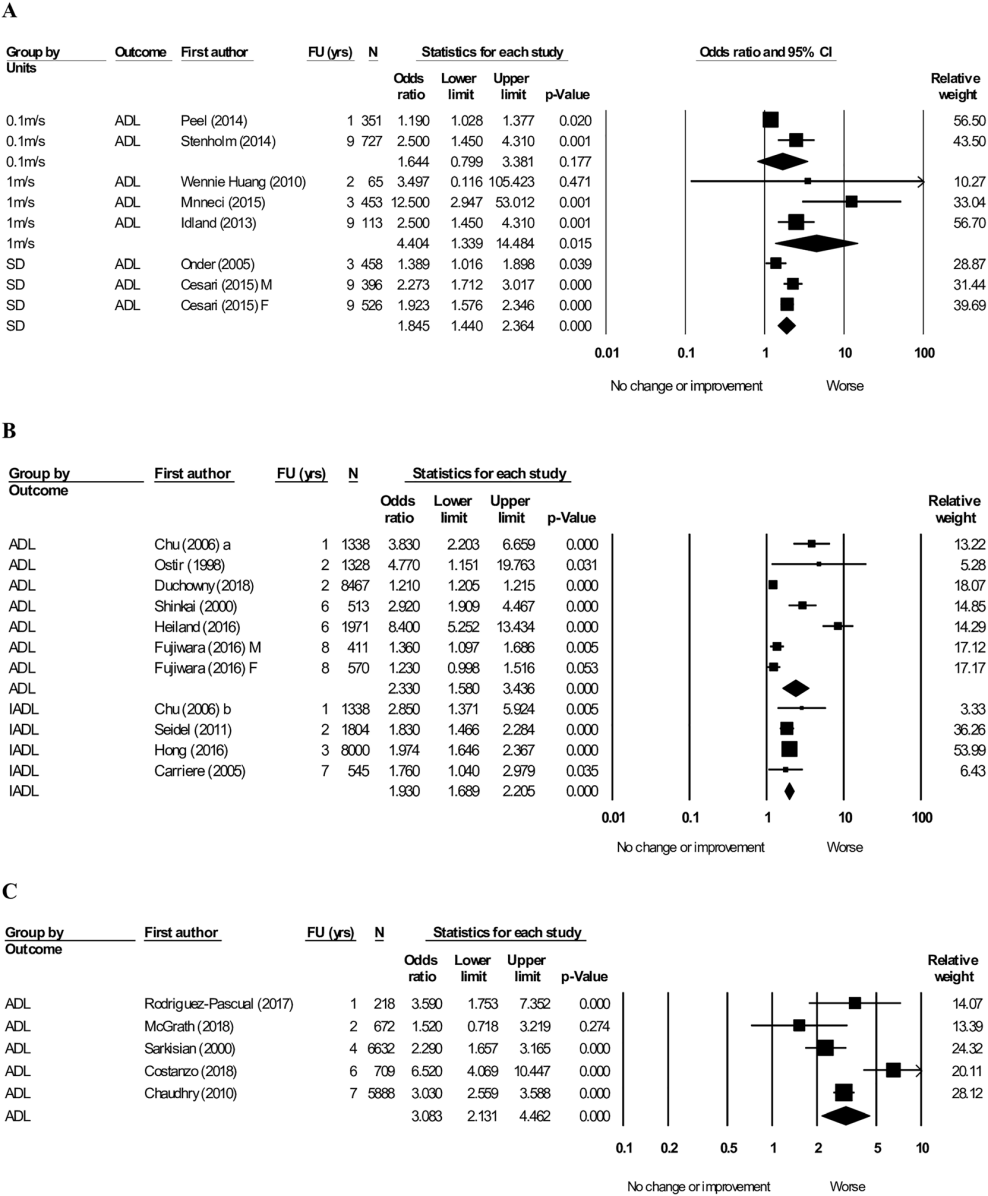
Forest plot showing the association between gait speed with activity of daily living (ADL) and instrumental activity of daily living (IADL) at follow‐up. (A) Gait (per unit lower), heterogeneity (*I* ^2^): 0.1 m/s = 85. 1.0 m/s = 52.1. SD = 62.8. (B) Gait (low vs. high), heterogeneity (*I* ^2^): ADL = 94.2. IADL = 0.0. (C) Gait (lowest quintile vs. upper four quintiles), heterogeneity (*I* ^2^) = 75.7.

Seven articles were pooled in a meta‐analysis demonstrating the association of gait speed (per unit increase) with ADL (*Figure* 5A). After subgrouping for a lower unit (per 0.1 m/s, per 1 m/s and per SD) in gait speed, a lower gait speed of 0.1 m/s^76^, ^96^ was not associated with worsening ADL (OR = 1.64, 95% CI: 0.80–3.38, *I* ^2^ = 85.0) while a lower gait speed of 1.0 m/s^63^, ^72^, ^102^ and 1 SD^38^, ^74^ was associated with worsening ADL (OR = 4.40, 95% CI: 1.34–14.48, *I* ^2^ = 52.1; OR = 1.85, 95% CI: 1.44–2.36, *I* ^2^ = 62.8). Six articles^41^, ^49^, ^52^, ^58^, ^75^, ^93^ pooling gait speed (low vs. high) with ADL favoured worsening in ADL (OR = 2.33, 95% CI: 1.58–3.44, *I* ^2^ = 94.2) (*Figure* 5B). Four articles^37^, ^41^, ^62^, ^90^ evaluating the association between gait speed (low vs. high) with IADL demonstrated worsening in IADL (OR = 1.93, 95% CI: 1.69–2.21, *I* ^2^ = 0.0) (*Figure* 5B). Five articles^40^, ^44^, ^71^, ^82^, ^87^ were pooled comparing the lowest quintile for gait speed with the upper four quintiles with ADL demonstrated an association in worsening ADL (OR = 3.08, 95% CI: 2.13–4.46, *I* ^2^ = 75.7) (*Figure* 5C).

**Figure 6:**
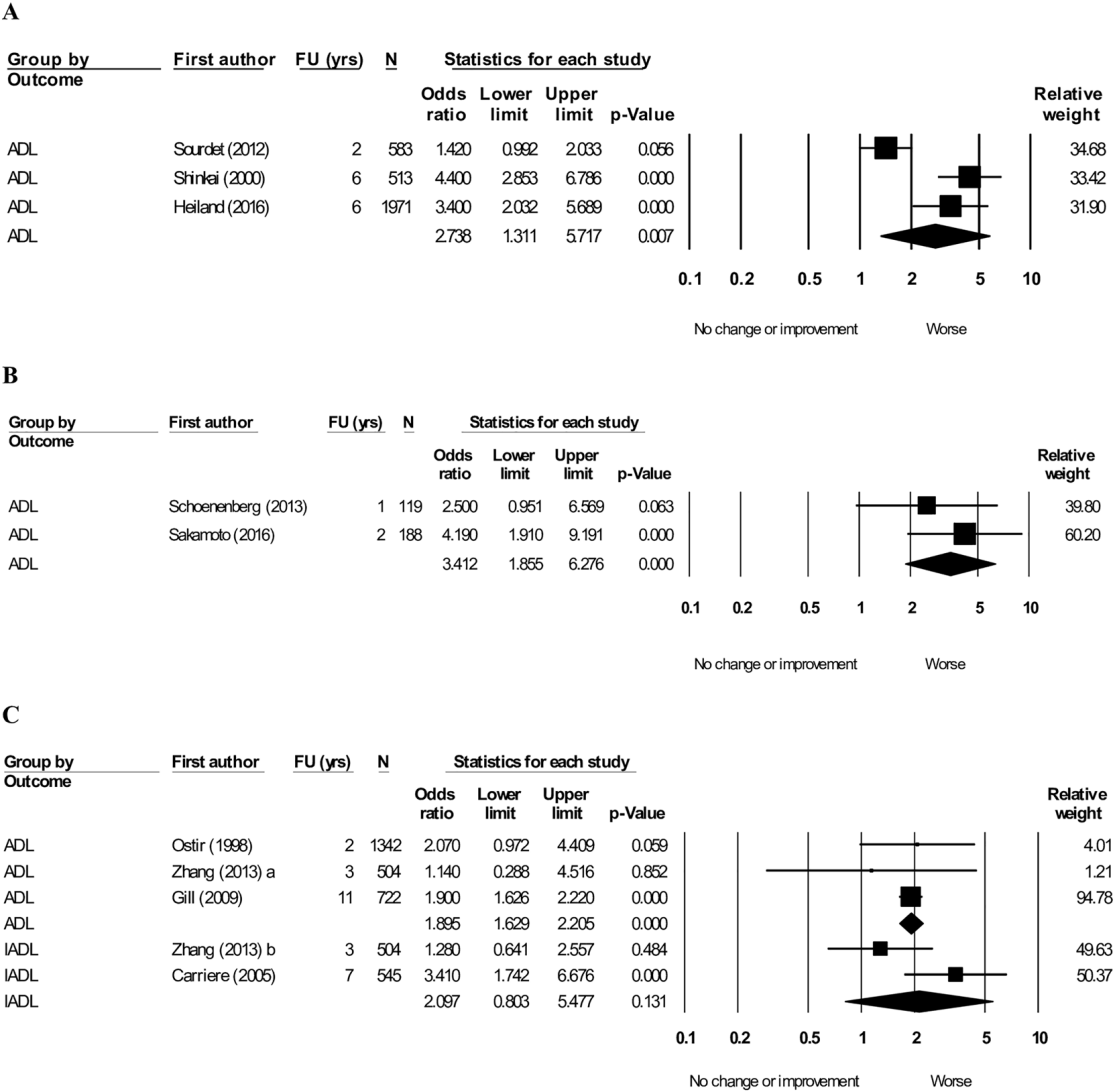
Forest plot showing the association between other physical performance measures with activity of daily living (ADL) at follow‐up. (A) One leg balance time (low vs. high), heterogeneity (*I* ^2^) = 88.5. (B) Timed up and go (slow vs. fast), heterogeneity (*I* ^2^) = 41.6. (C) Chair stand test time (slow vs. fast), heterogeneity (*I* ^2^): ADL = 0.0. Instrumental activity of daily living (IADL) = 74.8. Articles that reported both ADL and IADL were denoted a and b.

Three articles^58^, ^93^, ^95^ were pooled demonstrating a strong association between one leg balance (low vs. high) and a decline in ADL (OR = 2.74, 95% CI: 1.31–5.72, *I* ^2^ = 88.5) (*Figure* 6A). Two articles^84^, ^89^ reported timed up and go (slow vs. fast) with ADL (*Figure* 6B). Slow timed up and go favoured worsening in ADL (OR = 3.41, 95% CI: 1.86–6.28, *I* ^2^ = 41.6). Three articles^55^, ^75^, ^103^ explored the effect of chair stand test time (slow vs. fast) with ADL (*Figure* 6B). Slow chair stand test favoured worsening in ADL (OR = 1.90, 95% CI: 1.63–2.21, *I* ^2^ = 0.0). Two articles^37^, ^103^ were pooled exploring the effect of chair stand test time (slow vs. fast) was not associated with worsening IADL (OR = 2.10, 95% CI: 0.80–5.48, *I* ^2^ = 74.8) (*Figure* 6C).

All meta‐analyses were ordered by study follow‐up duration from shortest to longest. No pattern was present based on follow‐up duration in all meta‐analyses.

## Discussion

Muscle mass was associated with the development of IADL dependence. Muscle strength and physical performance were associated with the development of ADL and IADL dependence at follow‐up in older adults.

### Muscle mass

Surprisingly, muscle mass was associated with the development of new IADL dependence, but not ADL dependence at follow‐up in this meta‐analysis. While the association between muscle mass and ADL was not statistically significant, perhaps because of only two studies being included, a trend suggested that it was a clinically significant predictor of worsening ADL. The Concord Health and Ageing in Men Project reported that low muscle mass and sarcopenic obesity were significantly associated with worsening ADL and IADL.^60^ Additionally, a prospective study reported that low fat free mass was associated with functional disability.^36^ It has been previously hypothesized that muscle mass plays an important role in the loss of ADL with increasing age 106. Loss of muscle mass could be explained by a variety of different factors including the loss of innervation from alpha‐motor neurons 107, reduced dietary protein intake 108, 109, and less physical activity 110. Individuals with lower muscle mass have more difficulty in performing ADL and IADL 111, 112. Furthermore, it has been suggested that the infiltration of fat into the muscle is a risk factor for low muscle mass, which is associated with worsening ADL and IADL 111. Prior studies exploring the effect of training on muscle mass demonstrate its effectiveness in increasing performance and preservation of ADL and IADL 113, 114. Muscle mass has also been shown to improve in response to resistance exercise training and nutritional interventions, even in frail populations 109, 113, 115, 116, 117.

### Muscle strength

The predictive ability of muscle strength at baseline of ADL and IADL decline is consistent with a previous study consisting of 6089 participants, which suggested that having higher muscle strength was protective against the onset of future disability.^80^ However, the discrepancies in measures and cut‐offs and thus a lack of consensus in measuring muscle strength. Handgrip strength has been used as a measure of upper limb strength in older populations.^80^, ^118^ However handgrip strength should not be used as an approximation of overall muscle strength in older adults because of the variation between individuals and the variation in muscle groups within the same individual 119. Although a strong association between muscle mass and muscle strength exists 120, 121, 122, muscle strength may better predict worsening ADL and IADL as muscle mass can also be influenced by factors like disease, muscle use, and muscle morphology 123.

It has previously been hypothesized that there is a minimal amount of muscle strength required to complete ADL 124. Handgrip strength declines by 0.06 kg per year up to the age of 50 with an even steeper decline of 0.37 kg per year after the age of 50 years 125. In healthy older adults, large changes in muscle strength have little effect on ADL while small changes in a frail population have a more profound effect 124. This suggests that the completion of ADL and IADL requires a minimum threshold of strength and that higher muscle strength provides individuals with a protective reserve against the development of ADL and IADL dependence.^14^ Thus, individuals with lower muscle strength, and therefore a lower protective reserve, are at higher risk of developing new ADL and IADL dependence at follow‐up.^14^

The prevalence of ADL and IADL dependence increases with age, muscle strength amongst older adults has been shown to decrease.^126^, ^127^, ^128^, ^129^ Gender has also been suggested to influence muscle strength as hospitalized older adults demonstrated an association between handgrip strength with ADL and IADL in male patients, but not female patients 112. Individuals with high muscle strength at baseline are also likely to preserve their higher handgrip strength at follow‐up 129. It would be expected that the association is stronger with longer follow‐up duration. However, this was not observed in our meta‐analysis. A reason this could be due to the different baseline ages and follow‐up duration between studies. Given that muscle mass decreases at a slower rate compared with muscle strength, it may be easier to preserve and maintain muscle mass, which can also prevent the decline in muscle strength.^130^

Muscle quality, which refers to the muscle strength or power per unit of muscle mass,^131^ has been associated with worse ADL in a previous cross‐sectional study.^10^ As previous studies support the assumption that muscle strength decline occurs faster than muscle mass, it also suggests that muscle quality has likewise declined 130. Physical performance measured by gait speed has previously shown that a higher gait speed to be associated with higher muscle quality in older adults 132, 133. Currently, no studies have explored the association between muscle quality and worsening ADLs; however, as muscle quality has been shown to decline with age 134, 135, 136, it can be expected to observe similar results as muscle mass, strength, and physical performance.

### Physical performance

Lower SPPB scores were associated with worsening ADL at follow‐up. The SPPB encompasses three assessments that require strength, balance, dexterity, and cognitive control, which captures functional elements that are required for the completion of ADL and IADL 137, 138, 139. Lower extremity physical performance measured by the SPPB was associated with worse ADL. Furthermore, a difference in score of 1 point on the SPPB was also significantly associated with worse ADLs as shown in this review and prior research 138. Lower SPPB scores are a reflection of impaired skeletal muscle function and structural changes associated with chronic inflammatory processes 140, as well as neurological pathologies that impair gait and balance 141 that are thought to mediate the development of ADL and IADL dependence 142. High heterogeneity was observed between studies that evaluated the SPPB as a continuous variable compared with a dichotomous variable (low vs. high). The findings from this review were consistent with a previous systematic review, where the SPPB was predictive of long‐term disability.^143^

Gait speed measured by a 4 m walk test is a component of the SPPB and is often used in clinical settings to identify individuals at high risk of adverse health outcomes^144^ and assist in the diagnosis of sarcopenia.^145^ Gait speed is both a simple and highly reproducible measure of physical performance and is comparable with the SPPB as a predictor of ADL dependence.^56^, ^146^ In community‐dwelling outpatients, the association between gait sped with ADL and IADL was stronger than its association with the other sarcopenia diagnosis criteria.^147^ While having a higher 0.1 m/s gait speed was not statistically significant in this study, all other analyses showed a lower gait speed favoured a worsening ADL and/or IADL. One study, which evaluated 27 200 community‐dwelling older adults for gait speed, demonstrated its predictive value on the development of disability.^148^ Other studies reported different cut‐offs for slow and fast gait speed, but no single threshold was evident with the incidence of disability.^148^ Therapies or preventative interventions targeted at improving or maintaining gait speed should be considered amongst older adults, as these changes are reflective of the progression and development of ADL and IADL dependence.^149^, ^150^ The findings of this meta‐analysis also resonate with a previous systematic review, which demonstrated that slow gait speed was associated with worsening ADL in older populations.^15^, ^151^

One leg balance was significantly associated with worsening ADL in the follow‐up. The importance of balance is well described controlling both static and dynamic posture while performing a variety of daily activities,^152^, ^153^ such that it has been used as a predictor of high‐risk individuals prone to falls.^154^ Although one previous study reported no statistically significant association between one leg balance and ADL dependence, the three studies included in this meta‐analysis demonstrated a strong association between low one leg balance time and worsening ADLs.^155^ Timed up and go is a measure of walking, balance, strength, and cognition.^156^ A descriptive meta‐analysis exploring the cut‐off times of the timed up and go test reported cut‐off values of 8.1, 9.2, and 11.3 s for those aged 60–69, 70–79, and 80–99 years old, respectively.^157^ All studies included in the meta‐analysis^84^, ^89^, ^91^ had greater cut‐offs for a slow timed up and go time, potentially underestimating the pooled effect size. Timed up and go had a smaller effect size but less heterogeneity in predicting worsening ADLs compared with one leg balance in this meta‐analysis. Prior research comparing different measures of balance reported timed up and go as being a better predictor of ADL in older the community‐dwelling population.^158^

The chair stand test was significantly associated with worsening ADL but not IADL. Chair stand test has been used as a measure of lower body strength in older adults in community‐dwelling older adults and as part of the SPPB.^11^, ^159^ A previous study reported that 22% of community‐dwelling older individuals are unable to complete the chair stand test (5 to 10 rises) without the use of hands and/or arms.^11^ The 30 s chair stand test may be a better protocol as it is more reliable given that some individuals are unable to complete standard protocols and the floor effect.^159^ Surprisingly, no studies included in the meta‐analysis reported the 30 s protocol; only the 5× chair stand test was used. High heterogeneity was observed in IADL perhaps because of the different cut‐off points used (11.2 s^103^ and 13 s^160^) although this was not the case for ADLs.

### Strength and limitations

This systematic review and meta‐analysis explored muscle mass, muscle strength, and physical performance as predictors for ADL and IADL rather than limiting to just a single measure. By evaluating the effects of all three measures, this review presented the most detailed assessment of this topic. To reduce the risk of reverse causation when interpreting the associations reported, only prospective studies were included. There are also limitations to this review. Not all studies were pooled into a meta‐analysis because of differences in measures and cut‐offs of muscle mass, muscle strength, and physical performance, the use of different statistical analyses, or the lack of data required to calculate an OR. Studies that were pooled were also reporting univariate and multivariate analyses, adjusting for different confounders that were inconsistent between studies that may have further led to an over or under estimation of the effect sizes. Studies that did not have a primary aim of exploring the association between baseline muscle measures with ADL and/or IADL may have used less standardized procedures. The follow‐up duration between studies varied significantly, which may have impacted the results.

### Future recommendations

There were few pre‐existing studies that explored the association between baseline muscle mass and ADL and/or IADL. Studies should continue to investigate the association of muscle mass alone with ADL and/or IADL. Future studies should also explore the association of muscle quality with ADL and/or IADL. Interventions tailored for specifically increasing muscle measures should be developed to prevent individuals from having worsening ADL and/or IADL in their future. Interventions should be developed with the focus of preserving or increasing muscle measures as we age.

## Conclusions

This study quantified the current research available between muscle measures and (I)ADL. Muscle mass is predictive of ADL and IADL decline whereas muscle strength and physical performance are predictive of both ADL and/or IADL decline. Future studies should continue to develop and improve interventions to preserve and improve muscle measures to prevent ADL and IADL dependence.

## Conflict of Interest

Daniel X. M. Wang, Jessica Yao, Yasar Zirek, Esmee M. Reijnierse, and Andrea B. Maier declare that they have no conflict of interest.

## Supporting information


**Table S1.** Search Strategy

**Data S2.** Newcastle–Ottawa Scale

**Table S3**. Breakdown of the ADL components in articles that created their own questionnaire

## References

[1] Stuck AE , Walthert JM , Nikolaus T , Bula CJ , Hohmann C , Beck JC . Risk factors for functional status decline in community‐living elderly people: a systematic literature review. Soc Sci Med. 1999;48:445–469. 10075171 10.1016/s0277-9536(98)00370-0

[2] Millan‐Calenti JC , Tubio J , Pita‐Fernandez S , Gonzalez‐Abraldes I , Lorenzo T , Fernandez‐Arruty T , et al. Prevalence of functional disability in activities of daily living (ADL), instrumental activities of daily living (IADL) and associated factors, as predictors of morbidity and mortality. Arch Gerontol Geriatr. 2010;50:306–310. 19520442 10.1016/j.archger.2009.04.017

[3] Lawton MP , Brody EM . Assessment of older people: self‐maintaining and instrumental activities of daily living. The gerontologist. 1969;9:179–186. 5349366

[4] Velazquez Alva Mdel C , Irigoyen Camacho ME , Delgadillo Velazquez J , Lazarevich I . The relationship between sarcopenia, undernutrition, physical mobility and basic activities of daily living in a group of elderly women of Mexico City. Nutricion hospitalaria. 2013;28:514–521. 23822706 10.3305/nh.2013.28.2.6180

[5] Sonn U , Asberg KH . Assessment of activities of daily living in the elderly. A study of a population of 76‐year‐olds in Gothenburg, Sweden. Scand J Rehabil Med. 1991;23:193–202. 1785028

[6] Millan‐Calenti JC , Tubío J , Pita‐Fernández S , González‐Abraldes I , Lorenzo T , Fernandez‐Arruty T , et al. Prevalence of functional disability in activities of daily living (ADL), instrumental activities of daily living (IADL) and associated factors, as predictors of morbidity and mortality. Arch Gerontol Geriatr. 2010;50:306–310. 19520442 10.1016/j.archger.2009.04.017

[7] Berlau DJ , Corrada MM , Kawas C . The prevalence of disability in the oldest‐old is high and continues to increase with age: findings from The 90+ Study. Int J Geriatr Psychiatry. 2009;24:1217–1225. 19259982 10.1002/gps.2248 PMC2783224

[8] Cruz‐Jentoft AJ , Baeyens JP , Bauer JM , Boirie Y , Cederholm T , Landi F , et al. Sarcopenia: European consensus on definition and diagnosis: report of the European Working Group on sarcopenia in older people. Age and ageing. 2010;39:412–423. 20392703 10.1093/ageing/afq034 PMC2886201

[9] Janssen I , Heymsfield SB , Ross R . Low relative skeletal muscle mass (sarcopenia) in older persons is associated with functional impairment and physical disability. Journal of the American Geriatrics Society. 2002;50:889–896. 12028177 10.1046/j.1532-5415.2002.50216.x

[10] Hairi NN , Cumming RG , Naganathan V , Handelsman DJ , Le Couteur DG , Creasey H , et al. Loss of muscle strength, mass (sarcopenia), and quality (specific force) and its relationship with functional limitation and physical disability: the Concord Health and Ageing in Men Project. J Am Geriatr Soc. 2010;58:2055–2062. 21054284 10.1111/j.1532-5415.2010.03145.x

[11] Guralnik JM , Simonsick EM , Ferrucci L , Glynn RJ , Berkman LF , Blazer DG , et al. A short physical performance battery assessing lower extremity function: association with self‐reported disability and prediction of mortality and nursing home admission. J Gerontol. 1994;49:M85–M94. 8126356 10.1093/geronj/49.2.m85

[12] Hortobagyi T , Mizelle C , Beam S , DeVita P . Old adults perform activities of daily living near their maximal capabilities. J Gerontol A Biol Sci Med Sci. 2003;58:M453–M460. 12730256 10.1093/gerona/58.5.m453

[13] Gill TM , Robison JT , Tinetti ME . Predictors of recovery in activities of daily living among disabled older persons living in the community. J Gen Intern Med. 1997;12:757–762. 9436895 10.1046/j.1525-1497.1997.07161.x PMC1497202

[14] Rantanen T . Muscle strength, disability and mortality. Scand J Med Sci Sports. 2003;13:3–8. 12535311 10.1034/j.1600-0838.2003.00298.x

[15] Vermeulen J , Neyens JC , van Rossum E , Spreeuwenberg MD , de Witte LP . Predicting ADL disability in community‐dwelling elderly people using physical frailty indicators: a systematic review. BMC geriatrics. 2011;11:33. 21722355 10.1186/1471-2318-11-33 PMC3142492

[16] Jonkman NH , Del Panta V , Hoekstra T , Colpo M , van Schoor NM , Bandinelli S , et al. Predicting trajectories of functional decline in 60‐ to 70‐year‐old people. Gerontology. 2018;64:212–221. 29232671 10.1159/000485135 PMC5969068

[17] Velazquez Alva MC , Irigoyen Camacho ME , Delgadillo Velazquez J , Lazarevich I . The relationship between sarcopenia, undernutrition, physical mobility and basic activities of daily living in a group of elderly women of Mexico City. Nutricion hospitalaria. 2013;28:514–521. 23822706 10.3305/nh.2013.28.2.6180

[18] Taekema DG , Gussekloo J , Maier AB , Westendorp RGJ , de Craen AJM . Handgrip strength as a predictor of functional, psychological and social health. A prospective population‐based study among the oldest old. Age Ageing. 2010;39:331–337. 20219767 10.1093/ageing/afq022

[19] Moher D , Liberati A , Tetzlaff J , Altman DG . Preferred Reporting Items for Systematic Reviews and Meta‐analyses: the PRISMA statement. Ann Intern Med. 2009;151:264–269. 19622511 10.7326/0003-4819-151-4-200908180-00135

[20] Stang A . Critical evaluation of the Newcastle–Ottawa Scale for the assessment of the quality of nonrandomized studies in meta‐analyses. Eur J Epidemiol. 2010;25:603–605. 20652370 10.1007/s10654-010-9491-z

[21] Hermont AP , Oliveira PA , Martins CC , Paiva SM , Pordeus IA , Auad SM . Tooth erosion and eating disorders: a systematic review and meta‐analysis. PLoS One. 2014;9:e111123. 25379668 10.1371/journal.pone.0111123 PMC4224381

[22] Fleiss JL . The statistical basis of meta‐analysis. Stat Methods Med Res. 1993;2:121–145. 8261254 10.1177/096228029300200202

[23] Higgins JP , Thompson SG . Quantifying heterogeneity in a meta‐analysis. Stat Med. 2002;21:1539–1558. 12111919 10.1002/sim.1186

[24] Abete P , Basile C , Bulli G , Curcio F , Liguori I , Della‐Morte D , et al. The Italian version of the “frailty index” based on deficits in health: a validation study. Aging Clin Exp Res. 2017;29:913–926. 28688080 10.1007/s40520-017-0793-9

[25] Al Snih S , Markides KS , Ottenbacher KJ , Raji MA . Hand grip strength and incident ADL disability in elderly Mexican Americans over a seven‐year period. Aging Clin Exp Res. 2004;16:481–486. 15739601 10.1007/BF03327406

[26] Albert SM , Bear‐Lehman J , Anderson SJ . Declines in mobility and changes in performance in the instrumental activities of daily living among mildly disabled community‐dwelling older adults. J Gerontol A Biol Sci Med Sci. 2015;70:71–77. 24952575 10.1093/gerona/glu088 PMC4296164

[27] Alexandre TS , Corona LP , Nunes DP , Santos JLF , Duarte YAO , Lebrao ML . Gender differences in incidence and determinants of disability in activities of daily living among elderly individuals: SABE study. Arch Gerontol Geriatr. 2012;55:431–437. 22546518 10.1016/j.archger.2012.04.001

[28] Amigues I , Schott A‐M , Amine M , Gelas‐Dore B , Veerabudun K , Paillaud E , et al. Low skeletal muscle mass and risk of functional decline in elderly community‐dwelling women: the prospective EPIDOS study. J Am Med Dir Assoc. 2013;14:352–357. 23333310 10.1016/j.jamda.2012.12.002

[29] Arnau A , Espaulella J , Serrarols M , Canudas J , Formiga F , Ferrer M . Risk factors for functional decline in a population aged 75 years and older without total dependence: a one‐year follow‐up. Arch Gerontol Geriatr. 2016;65:239–247. 27131227 10.1016/j.archger.2016.04.002

[30] Artaud F , Singh‐Manoux A , Dugravot A , Tzourio C , Elbaz A . Decline in fast gait speed as a predictor of disability in older adults. J Am Geriatr Soc. 2015;63:1129–1136. 26096387 10.1111/jgs.13442

[31] Basic D , Ni Chroinin D , Conforti D , Shanley C . Predictors on admission of functional decline among older patients hospitalised for acute care: a prospective observational study. Australas J Ageing. 2017;36:E57–E63. 28856791 10.1111/ajag.12458

[32] Baumgartner RN , Wayne SJ , Waters DL , Janssen I , Gallagher D , Morley JE . Sarcopenic obesity predicts instrumental activities of daily living disability in the elderly. Obes Res. 2004;12:1995–2004. 15687401 10.1038/oby.2004.250

[33] Beauchamp MK , Jette AM , Ward RE , Kurlinski LA , Kiely D , Latham NK , et al. Predictive validity and responsiveness of patient‐reported and performance‐based measures of function in the Boston RISE study. J Gerontol A Biol Sci Med Sci. 2015;70:616–622. 25512569 10.1093/gerona/glu227 PMC4400398

[34] Beloosesky Y , Weiss A , Manasian M , Salai M . Handgrip strength of the elderly after hip fracture repair correlates with functional outcome. Disabil Rehabil. 2010;32:367–373. 20025431 10.3109/09638280903168499

[35] Bianchi L , Ferrucci L , Cherubini A , Maggio M , Bandinelli S , Savino E , et al. The predictive value of the EWGSOP definition of sarcopenia: results from the InCHIANTI study. J Gerontol A Biol Sci Med Sci. 2016;71:259–264. 26333772 10.1093/gerona/glv129 PMC4723661

[36] Broadwin J , Goodman‐Gruen D , Slymen D . Ability of fat and fat‐free mass percentages to predict functional disability in older men and women. J Am Geriatr Soc. 2001;49:1641–1645. 11843997 10.1046/j.1532-5415.2001.t01-1-49273.x

[37] Carriere I , Colvez A , Favier F , Jeandel C , Blain H , Epidos study g . Hierarchical components of physical frailty predicted incidence of dependency in a cohort of elderly women. J Clin Epidemiol. 2005;58:1180–1187. 16223662 10.1016/j.jclinepi.2005.02.018

[38] Cesari M , Rolland Y , Abellan Van Kan G , Bandinelli S , Vellas B , Ferrucci L . Sarcopenia‐related parameters and incident disability in older persons: results from the “invecchiare in Chianti” study. J Gerontol A Biol Sci Med Sci. 2015;70:457–463. 25320055 10.1093/gerona/glu181 PMC4375415

[39] Chan OYA , van Houwelingen AH , Gussekloo J , Blom JW , den Elzen WPJ . Comparison of quadriceps strength and handgrip strength in their association with health outcomes in older adults in primary care. Age (Dordr). 2014;36:9714. 25280549 10.1007/s11357-014-9714-4 PMC4185022

[40] Chaudhry SI , McAvay G , Ning Y , Allore HG , Newman AB , Gill TM . Geriatric impairments and disability: the cardiovascular health study. J Am Geriatr Soc. 2010;58:1686–1692. 20863328 10.1111/j.1532-5415.2010.03022.x PMC2946108

[41] Chu L‐W , Chiu AYY , Chi I . Impact of falls on the balance, gait, and activities of daily living functioning in community‐dwelling Chinese older adults. J Gerontol A Biol Sci Med Sci. 2006;61:399–404. 16611708 10.1093/gerona/61.4.399

[42] Cooper R , Huisman M , Kuh D , Deeg DJH . Do positive psychological characteristics modify the associations of physical performance with functional decline and institutionalization? Findings from the longitudinal aging study Amsterdam. J Gerontol B Psychol Sci Soc Sci. 2011;66:468–477. 21743041 10.1093/geronb/gbr049 PMC3132268

[43] Corsonello A , Lattanzio F , Pedone C , Garasto S , Laino I , Bustacchini S , et al. Prognostic significance of the short physical performance battery in older patients discharged from acute care hospitals. Rejuvenation Res. 2012;15:41–48. 22004280 10.1089/rej.2011.1215 PMC3283437

[44] Costanzo L , Pedone C , Cesari M , Ferrucci L , Bandinelli S , Antonelli Incalzi R . Clusters of functional domains to identify older persons at risk of disability. Geriatr Gerontol Int. 2018;18:685–691. 29282845 10.1111/ggi.13226 PMC5934311

[45] den Ouden MEM , Schuurmans MJ , Brand JS , Arts IEMA , Mueller‐Schotte S , van der Schouw YT . Physical functioning is related to both an impaired physical ability and ADL disability: a ten year follow‐up study in middle‐aged and older persons. Maturitas. 2013;74:89–94. 23159191 10.1016/j.maturitas.2012.10.011

[46] Denkinger MD , Igl W , Jamour M , Bader A , Bailer S , Lukas A , et al. Does functional change predict the course of improvement in geriatric inpatient rehabilitation? Clin Rehabil. 2010;24:463–470. 20354056 10.1177/0269215509353269

[47] Di Monaco M , Castiglioni C , De Toma E , Gardin L , Giordano S , Tappero R . Handgrip strength is an independent predictor of functional outcome in hip‐fracture women: a prospective study with 6‐month follow‐up. Medicine (Baltimore). 2015;94:e542. 25674760 10.1097/MD.0000000000000542 PMC4602757

[48] Donoghue OA , Savva GM , Cronin H , Kenny RA , Horgan NF . Using timed up and go and usual gait speed to predict incident disability in daily activities among community‐dwelling adults aged 65 and older. Arch Phys Med Rehabil. 2014;95:1954–1961. 24977931 10.1016/j.apmr.2014.06.008

[49] Duchowny KA , Clarke PJ , Peterson MD . Muscle weakness and physical disability in older Americans: longitudinal findings from the U.S. Health and Retirement Study. J Nutr Health Aging. 2018;22:501–507. 29582889 10.1007/s12603-017-0951-y PMC6472265

[50] Fantin F , Francesco VD , Fontana G , Zivelonghi A , Bissoli L , Zoico E , et al. Longitudinal body composition changes in old men and women: interrelationships with worsening disability. J Gerontol A Biol Sci Med Sci. 2007;62:1375–1381. 18166688 10.1093/gerona/62.12.1375

[51] Femia EE , Zarit SH , Johansson B . Predicting change in activities of daily living: a longitudinal study of the oldest old in Sweden. J Gerontol B Psychol Sci Soc Sci. 1997;52:P294–P302. 9403518 10.1093/geronb/52b.6.p294

[52] Fujiwara Y , Shinkai S , Kobayashi E , Minami U , Suzuki H , Yoshida H , et al. Engagement in paid work as a protective predictor of basic activities of daily living disability in Japanese urban and rural community‐dwelling elderly residents: an 8‐year prospective study. Geriatr Gerontol Int. 2016;16:126–134. 25612931 10.1111/ggi.12441

[53] Giampaoli S , Ferrucci L , Cecchi F , Noce CL , Poce A , Dima F , et al. Hand‐grip strength predicts incident disability in non‐disabled older men. Age Ageing. 1999;28:283–288. 10475865 10.1093/ageing/28.3.283

[54] Gill TM , Williams CS , Richardson ED , Tinetti ME . Impairments in physical performance and cognitive status in predisposing factors for functional dependence among nondisabled older persons. J Gerontol A Biol Sci Med Sci. 1996;51A:M283–M288. 10.1093/gerona/51a.6.m283 8914500

[55] Gill TM , Murphy TE , Barry LC , Allore HG . Risk factors for disability subtypes in older persons. J Am Geriatr Soc. 2009;57:1850–1855. 19694870 10.1111/j.1532-5415.2009.02443.x PMC2782909

[56] Guralnik JM , Ferrucci L , Pieper CF , Leveille SG , Markides KS , Ostir GV , et al. Lower extremity function and subsequent disability: consistency across studies, predictive models, and value of gait speed alone compared with the short physical performance battery. J Gerontol A Biol Sci Med Sci. 2000;55:M221–M231. 10811152 10.1093/gerona/55.4.m221 PMC12149745

[57] Hansen K , Mahoney J , Palta M . Risk factors for lack of recovery of ADL independence after hospital discharge. J Am Geriatr Soc. 1999;47:360–365. 10078901 10.1111/j.1532-5415.1999.tb03002.x

[58] Heiland EG , Welmer A‐K , Wang R , Santoni G , Angleman S , Fratiglioni L , et al. Association of mobility limitations with incident disability among older adults: a population‐based study. Age Ageing. 2016;45:812–819. 27126329 10.1093/ageing/afw076

[59] Hirani V , Blyth F , Naganathan V , Le Couteur DG , Seibel MJ , Waite LM , et al. Sarcopenia is associated with incident disability, institutionalization, and mortality in community‐dwelling older men: the Concord Health and Ageing in Men Project. J Am Med Dir Assoc. 2015;16:607–613. 25820131 10.1016/j.jamda.2015.02.006

[60] Hirani V , Naganathan V , Blyth F , Le Couteur DG , Seibel MJ , Waite LM , et al. Longitudinal associations between body composition, sarcopenic obesity and outcomes of frailty, disability, institutionalisation and mortality in community‐dwelling older men: the Concord Health and Ageing in Men Project. Age Ageing. 2017;46:413–420. 27932368 10.1093/ageing/afw214

[61] Hoeymans N , Feskens EJ , van den Bos GA , Kromhout D . Measuring functional status: cross‐sectional and longitudinal associations between performance and self‐report (Zutphen Elderly Study 1990‐1993). J Clin Epidemiol. 1996;49:1103–1110. 8826989 10.1016/0895-4356(96)00210-7

[62] Hong S , Kim S , Yoo J , Kim BS , Choi HR , Choi SE , et al. Slower gait speed predicts decline in instrumental activities of daily living in community‐dwelling elderly: 3‐year prospective finding from Living Profiles of Older People Survey in Korea. Journal of clinical gerontology & geriatrics. 2016;7:141–145.

[63] Idland G , Pettersen R , Avlund K , Bergland A . Physical performance as long‐term predictor of onset of activities of daily living (ADL) disability: a 9‐year longitudinal study among community‐dwelling older women. Arch Gerontol Geriatr. 2013;56:501–506. 23290919 10.1016/j.archger.2012.12.005

[64] Ishizaki T , Watanabe S , Suzuki T , Shibata H , Haga H . Predictors for functional decline among nondisabled older Japanese living in a community during a 3‐year follow‐up. J Am Geriatr Soc. 2000;48:1424–1429. 11083318 10.1111/j.1532-5415.2000.tb02632.x

[65] Janssen I . Influence of sarcopenia on the development of physical disability: the Cardiovascular Health Study. J Am Geriatr Soc. 2006;54:56–62. 16420198 10.1111/j.1532-5415.2005.00540.x

[66] Kempen GI , Ormel J . The impact of physical performance and cognitive status on subsequent ADL disability in low‐functioning older adults. Int J Geriatr Psychiatry. 1998;13:480–483. 9695038 10.1002/(sici)1099-1166(199807)13:7<480::aid-gps805>3.0.co;2-s

[67] Kozicka I , Kostka T . Handgrip strength, quadriceps muscle power, and optimal shortening velocity roles in maintaining functional abilities in older adults living in a long‐term care home: a 1‐year follow‐up study. Clin Interv Aging. 2016;11:739–747. 27307720 10.2147/CIA.S101043 PMC4887055

[68] Kwon S , Symons R , Yukawa M , Dasher N , Legner V , Flum DR . Evaluating the association of preoperative functional status and postoperative functional decline in older patients undergoing major surgery. Am Surg. 2012;78:1336–1344. 23265122 PMC4241019

[69] Legrand D , Vaes B , Mathei C , Adriaensen W , Van Pottelbergh G , Degryse J‐M . Muscle strength and physical performance as predictors of mortality, hospitalization, and disability in the oldest old. J Am Geriatr Soc. 2014;62:1030–1038. 24802886 10.1111/jgs.12840

[70] Lopez‐Teros T , Gutierrez‐Robledo LM , Perez‐Zepeda MU . Gait speed and handgrip strength as predictors of incident disability in Mexican older adults. J Frailty Aging. 2014;3:109–112. 27049903 10.14283/jfa.2014.10

[71] McGrath R , Robinson‐Lane SG , Peterson MD , Bailey RR , Vincent BM . Muscle strength and functional limitations: preserving function in older Mexican Americans. J Am Med Dir Assoc. 2018;19:391–398. 29371128 10.1016/j.jamda.2017.12.011 PMC6375488

[72] Minneci C , Mello AM , Mossello E , Baldasseroni S , Macchi L , Cipolletti S , et al. Comparative study of four physical performance measures as predictors of death, incident disability, and falls in unselected older persons: the insufficienza Cardiaca negli Anziani Residenti a Dicomano Study. J Am Geriatr Soc. 2015;63:136–141. 25597564 10.1111/jgs.13195

[73] Moen K , Ormstad H , Wang‐Hansen MS , Brovold T . Physical function of elderly patients with multimorbidity upon acute hospital admission versus 3 weeks post‐discharge. Disabil Rehabil. 2018;40:1280–1287. 28271911 10.1080/09638288.2017.1294211

[74] Onder G , Penninx BWJH , Ferrucci L , Fried LP , Guralnik JM , Pahor M . Measures of physical performance and risk for progressive and catastrophic disability: results from the Women's Health and Aging Study. J Gerontol A Biol Sci Med Sci. 2005;60:74–79. 15741286 10.1093/gerona/60.1.74

[75] Ostir GV , Markides KS , Black SA , Goodwin JS . Lower body functioning as a predictor of subsequent disability among older Mexican Americans. J Gerontol A Biol Sci Med Sci. 1998;53:M491–M495. 9823755 10.1093/gerona/53a.6.m491

[76] Peel NM , Navanathan S , Hubbard RE . Gait speed as a predictor of outcomes in post‐acute transitional care for older people. Geriatr Gerontol Int. 2014;14:906–910. 24666818 10.1111/ggi.12191

[77] Pisters MF , Veenhof C , van Dijk GM , Heymans MW , Twisk JWR , Dekker J . The course of limitations in activities over 5 years in patients with knee and hip osteoarthritis with moderate functional limitations: risk factors for future functional decline. Osteoarthritis Cartilage. 2012;20:503–510. 22330176 10.1016/j.joca.2012.02.002

[78] Purser JL , Weinberger M , Cohen HJ , Pieper CF , Morey MC , Li T , et al. Walking speed predicts health status and hospital costs for frail elderly male veterans. J Rehabil Res Dev. 2005;42:535–545. 16320148 10.1682/jrrd.2004.07.0087

[79] Rajan KB , Hebert LE , Scherr P , Dong X , Wilson RS , Evans DA , et al. Cognitive and physical functions as determinants of delayed age at onset and progression of disability. J Gerontol A Biol Sci Med Sci. 2012;67:1419–1426. 22539654 10.1093/gerona/gls098 PMC3636674

[80] Rantanen T , Guralnik JM , Foley D , Masaki K , Leveille S , Curb JD , et al. Midlife hand grip strength as a predictor of old age disability. JAMA. 1999;281:558–560. 10022113 10.1001/jama.281.6.558

[81] Rantanen T , Avlund K , Suominen H , Schroll M , Frandin K , Pertti E . Muscle strength as a predictor of onset of ADL dependence in people aged 75 years. Aging Clin Exp Res. 2002;14:10–15. 12475129

[82] Rodriguez‐Pascual C , Paredes‐Galan E , Ferrero‐Martinez A‐I , Gonzalez‐Guerrero J‐L , Hornillos‐Calvo M , Menendez‐Colino R , et al. The frailty syndrome is associated with adverse health outcomes in very old patients with stable heart failure: a prospective study in six Spanish hospitals. Int J Cardiol. 2017;236:296–303. 28215465 10.1016/j.ijcard.2017.02.016

[83] Rothman MD , Leo‐Summers L , Gill TM . Prognostic significance of potential frailty criteria. J Am Geriatr Soc. 2008;56:2211–2216. 19093920 10.1111/j.1532-5415.2008.02008.x PMC2782664

[84] Sakamoto R , Okumiya K , Ishine M , Wada T , Fujisawa M , Imai H , et al. Predictors of difficulty in carrying out basic activities of daily living among the old‐old: a 2‐year community‐based cohort study. Geriatr Gerontol Int. 2016;16:214–222. 25655001 10.1111/ggi.12462

[85] Sanchez‐Martinez M , Castell MV , Gonzalez‐Montalvo JI , De La Cruz JJ , Banegas JR , Otero A . Transitions in functional status of community dwelling older adults: impact of physical performance. depression and cognition. Eur Geriatr Med. 2016;7:111–116.

[86] Sanchez‐Rodriguez D , Marco E , Miralles R , Fayos M , Mojal S , Alvarado M , et al. Sarcopenia, physical rehabilitation and functional outcomes of patients in a subacute geriatric care unit. Arch Gerontol Geriatr. 2014;59:39–43. 24726179 10.1016/j.archger.2014.02.009

[87] Sarkisian CA , Liu H , Gutierrez PR , Seeley DG , Cummings SR , Mangione CM . Modifiable risk factors predict functional decline among older women: a prospectively validated clinical prediction tool. The Study of Osteoporotic Fractures Research Group. J Am Geriatr Soc. 2000;48:170–178. 10682946 10.1111/j.1532-5415.2000.tb03908.x

[88] Sarkisian CA , Liu H , Ensrud KE , Stone KL , Mangione CM . Correlates of attributing new disability to old age. Study of Osteoporotic Fractures Research Group. J Am Geriatr Soc. 2001;49:134–141. 11207866 10.1046/j.1532-5415.2001.49033.x

[89] Schoenenberger AW , Stortecky S , Neumann S , Moser A , Juni P , Carrel T , et al. Predictors of functional decline in elderly patients undergoing transcatheter aortic valve implantation (TAVI). Eur Heart J. 2013;34:684–692. 23008508 10.1093/eurheartj/ehs304

[90] Seidel D , Brayne C , Jagger C . Limitations in physical functioning among older people as a predictor of subsequent disability in instrumental activities of daily living. Age Ageing. 2011;40:463–469. 21609999 10.1093/ageing/afr054 PMC3114622

[91] Shimada H , Sawyer P , Harada K , Kaneya S , Nihei K , Asakawa Y , et al. Predictive validity of the classification schema for functional mobility tests in instrumental activities of daily living decline among older adults. Arch Phys Med Rehabil. 2010;91:241–246. 20159128 10.1016/j.apmr.2009.10.027

[92] Shimada H , Makizako H , Doi T , Tsutsumimoto K , Suzuki T . Incidence of disability in frail older persons with or without slow walking speed. J Am Med Dir Assoc. 2015;16:690–696. 25922120 10.1016/j.jamda.2015.03.019

[93] Shinkai S , Watanabe S , Kumagai S , Fujiwara Y , Amano H , Yoshida H , et al. Walking speed as a good predictor for the onset of functional dependence in a Japanese rural community population. Age Ageing. 2000;29:441–446. 11108417 10.1093/ageing/29.5.441

[94] Shinkai S , Kumagai S , Fujiwara Y , Amano H , Yoshida Y , Watanabe S , et al. Predictors for the onset of functional decline among initially non‐disabled older people living in a community during a 6‐year follow‐up. Geriatr Gerontol Int. 2003;3:S31–S39.

[95] Sourdet S , van Kan GA , Soto ME , Houles M , Cantet C , Nourhashemi F , et al. Prognosis of an abnormal one‐leg balance in community‐dwelling patients with Alzheimer's disease: a 2‐year prospective study in 686 patients of the REAL.FR study. J Am Med Dir Assoc. 2012;13: 407.e1‐6. 10.1016/j.jamda.2011.11.003 22227074

[96] Stenholm S , Guralnik JM , Bandinelli S , Ferrucci L . The prognostic value of repeated measures of lower extremity performance: should we measure more than once? J Gerontol A Biol Sci Med Sci. 2014;69:894–899. 24270061 10.1093/gerona/glt175 PMC4067114

[97] Takuhiro O , Yasuyo A , Yoshihito T , Satoshi M , Mitsuo K , Kazuhiko A , et al. Age‐specific risk factors for incident disability in activities of daily living among middle‐aged and elderly community‐dwelling Japanese women during an 8–9‐year follow up: The Hizen‐Oshima study. Geriatr Gerontol Int. 2017;17:1096–1101. 27401720 10.1111/ggi.12834

[98] Tanimoto Y , Watanabe M , Sun W , Tanimoto K , Shishikura K , Sugiura Y , et al. Association of sarcopenia with functional decline in community‐dwelling elderly subjects in Japan. Geriatr Gerontol Int. 2013;13:958–963. 23452074 10.1111/ggi.12037

[99] Terhorst L , Holm MB , Toto PE , Rogers JC . Performance‐based impairment measures as predictors of early‐stage activity limitations in community‐dwelling older adults. J Aging Health. 2017;29:880–892. 27166414 10.1177/0898264316648113

[100] Tinetti ME , Allore H , Araujo KL , Seeman T . Modifiable impairments predict progressive disability among older persons. J Aging Health. 2005;17:239–256. 15750053 10.1177/0898264305275176

[101] Volpato S , Cavalieri M , Sioulis F , Guerra G , Maraldi C , Zuliani G , et al. Predictive value of the short physical performance battery following hospitalization in older patients. J Gerontol A Biol Sci Med Sci. 2011;66:89–96. 20861145 10.1093/gerona/glq167 PMC3011958

[102] Wennie Huang W‐N , Perera S , VanSwearingen J , Studenski S . Performance measures predict onset of activity of daily living difficulty in community‐dwelling older adults. J Am Geriatr Soc. 2010;58:844–852. 20406319 10.1111/j.1532-5415.2010.02820.x PMC2909370

[103] Zhang F , Ferrucci L , Culham E , Metter EJ , Guralnik J , Deshpande N . Performance on five times sit‐to‐stand task as a predictor of subsequent falls and disability in older persons. J Aging Health. 2013;25:478–492. 23407343 10.1177/0898264313475813

[104] Zoico E , Di Francesco V , Mazzali G , Zivelonghi A , Volpato S , Bortolani A , et al. High baseline values of fat mass, independently of appendicular skeletal mass, predict 2‐year onset of disability in elderly subjects at the high end of the functional spectrum. Aging Clin Exp Res. 2007;19:154–159. 17446727 10.1007/BF03324682

[105] Raji MA , Kuo Y‐F , Snih SA , Markides KS , Peek MK , Ottenbacher KJ . Cognitive status, muscle strength, and subsequent disability in older Mexican Americans. J Am Geriatr Soc. 2005;53:1462–1468. 16137273 10.1111/j.1532-5415.2005.53457.x

[106] Evans WJ . What is sarcopenia? The Journals of Gerontology: Series A. 1995;50A:5–8. 10.1093/gerona/50a.special_issue.5 7493218

[107] Brown WF . A method for estimating the number of motor units in thenar muscles and the changes in motor unit count with ageing. Journal of Neurology, Neurosurgery &amp;amp; Psychiatry. 1972;35:845. 10.1136/jnnp.35.6.845 PMC494191 4647858

[108] Young VR . Amino acids and proteins in relation to the nutrition of elderly people. Age and ageing. 1990;19:S10–S24. 2220475 10.1093/ageing/19.suppl_1.s10

[109] Martin‐Cantero A RE , Gill BMT , Maier AB . Factors influencing the efficacy of nutritional interventions on muscle mass in older adults: a systematic review and meta‐analysis. [under review]. 10.1093/nutrit/nuaa064 PMC7876433 33031516

[110] Westerterp KR . Daily physical activity and ageing. Curr Opin Clin Nutr Metab Care. 2000;3:485–488. 11085835 10.1097/00075197-200011000-00011

[111] Visser M , Goodpaster BH , Kritchevsky SB , Newman AB , Nevitt M , Rubin SM , et al. Muscle mass, muscle strength, and muscle fat infiltration as predictors of incident mobility limitations in well‐functioning older persons. J Gerontol A Biol Sci Med Sci. 2005;60:324–333. 15860469 10.1093/gerona/60.3.324

[112] Meskers C , Reijnierse E , Numans S , Kruizinga R , Pierik V , van Ancum J , et al. Association of handgrip strength and muscle mass with dependency in (instrumental) activities of daily living in hospitalized older adults—the EMPOWER study. J Nutr Health Aging. 1–7. 10.1007/s12603-019-1170-5 PMC6399821 30820510

[113] Fiatarone MA , O'Neill EF , Ryan ND , Clements KM , Solares GR , Nelson ME , et al. Exercise training and nutritional supplementation for physical frailty in very elderly people. N Engl J Med. 1994;330:1769–1775. 8190152 10.1056/NEJM199406233302501

[114] Brown M , Holloszy JO . Effects of walking, jogging and cycling on strength, flexibility, speed and balance in 60‐ to 72‐year olds. Aging (Milano). 1993;5:427–434. 8161574 10.1007/BF03324197

[115] Sipilä S , Multanen J , Kallinen M , Era P , Suominen H . Effects of strength and endurance training on isometric muscle strength and walking speed in elderly women. Acta Physiol Scand Suppl. 1996;156:457–464. 10.1046/j.1365-201X.1996.461177000.x 8732251

[116] Frontera WR , Meredith CN , O'Reilly KP , Knuttgen HG , Evans WJ . Strength conditioning in older men: skeletal muscle hypertrophy and improved function. J Appl Physiol (1985). 1988;64:1038–1044. 3366726 10.1152/jappl.1988.64.3.1038

[117] Kamleh AA RE , Aarden JJ , Daly RM , Andrea BM . The optimal resistance exercise training program for older adults to increase muscle mass: a systematic review and meta‐analysis. [under review].

[118] Fried LP , Ettinger WH , Lind B , Newman AB , Gardin J . Physical disability in older adults: a physiological approach. Cardiovascular Health Study Research Group. J Clin Epidemiol. 1994;47:747–760. 10.1016/0895-4356(94)90172-4 7722588

[119] Yeung SS , Reijnierse EM , Trappenburg MC , Hogrel J‐Y , McPhee JS , Piasecki M , et al. Handgrip strength cannot be assumed a proxy for overall muscle strength. J Am Med Dir Assoc. 2018. 10.1016/j.jamda.2018.04.019 29935982

[120] Reed RL , Pearlmutter L , Yochum K , Meredith KE , Mooradian AD . The relationship between muscle mass and muscle strength in the elderly. J Am Geriatr Soc. 1991;39:555–561. 1805811 10.1111/j.1532-5415.1991.tb03592.x

[121] Frontera WR , Hughes VA , Lutz KJ , Evans WJ . A cross‐sectional study of muscle strength and mass in 45‐ to 78‐yr‐old men and women. J Appl Physiol (1985). 1991;71:644–650. 1938738 10.1152/jappl.1991.71.2.644

[122] Freilich RJ , Kirsner RL , Byrne E . Isometric strength and thickness relationships in human quadriceps muscle. Neuromuscul Disord. 1995;5:415–422. 7496175 10.1016/0960-8966(94)00078-n

[123] Larsson L , Grimby G , Karlsson J . Muscle strength and speed of movement in relation to age and muscle morphology. J Appl Physiol Respir Environ Exerc Physiol. 1979;46:451–456. 438011 10.1152/jappl.1979.46.3.451

[124] Buchner DM , Larson EB , Wagner EH , Koepsell TD , De Lateur BJ . Evidence for a non‐linear relationship between leg strength and gait speed. Age Ageing. 1996;25:386–391. 8921145 10.1093/ageing/25.5.386

[125] Beenakker KG , Ling CH , Meskers CG , de Craen AJ , Stijnen T , Westendorp RG , et al. Patterns of muscle strength loss with age in the general population and patients with a chronic inflammatory state. Ageing Res Rev. 2010;9:431–436. 20553969 10.1016/j.arr.2010.05.005 PMC7105185

[126] Koyano W , Shibata H , Nakazato K , Haga H , Suyama Y , Matsuzaki T . Prevalence of disability in instrumental activities of daily living among elderly Japanese. J Gerontol A Biol Sci Med Sci. 1988;43:S41–S45. 10.1093/geronj/43.2.s41 2964469

[127] Covinsky KE , Palmer RM , Fortinsky RH , Counsell SR , Stewart AL , Kresevic D , et al. Loss of independence in activities of daily living in older adults hospitalized with medical illnesses: increased vulnerability with age. J Am Geriatr Soc. 2003;51:451–458. 12657063 10.1046/j.1532-5415.2003.51152.x

[128] Kallman DA , Plato CC , Tobin JD . The role of muscle loss in the age‐related decline of grip strength: cross‐sectional and longitudinal perspectives. J Gerontol. 1990;45:M82–M88. 2335723 10.1093/geronj/45.3.m82

[129] Rantanen T , Masaki K , Foley D , Izmirlian G , White L , Guralnik JM . Grip strength changes over 27 yr in Japanese‐American men. J Appl Physiol (1985). 1998;85:2047–2053. 9843525 10.1152/jappl.1998.85.6.2047

[130] Goodpaster BH , Park SW , Harris TB , Kritchevsky SB , Nevitt M , Schwartz AV , et al. The loss of skeletal muscle strength, mass, and quality in older adults: the health. aging and body composition study. J Gerontol A Biol Sci Med Sci. 2006;61:1059–1064. 17077199 10.1093/gerona/61.10.1059

[131] Barbat‐Artigas S , Rolland Y , Zamboni M , Aubertin‐Leheudre M . How to assess functional status: a new muscle quality index. J Nutr Health Aging. 2012;16:67–77.2223800410.1007/s12603-012-0004-5

[132] Shin S , Valentine RJ , Evans EM , Sosnoff JJ . Lower extremity muscle quality and gait variability in older adults. Age Ageing. 2012;41:595–599. 22417983 10.1093/ageing/afs032

[133] Cawthon PM , Fox KM , Gandra SR , Delmonico MJ , Chiou C‐F , Anthony MS , et al. Do muscle mass, muscle density, strength, and physical function similarly influence risk of hospitalization in older adults? J Am Geriatr Soc. 2009;57:1411–1419. 19682143 10.1111/j.1532-5415.2009.02366.x PMC3269169

[134] Metter EJ , Lynch N , Conwit R , Lindle R , Tobin J , Hurley B . Muscle quality and age: cross‐sectional and longitudinal comparisons. J Gerontol A Biol Sci Med Sci. 1999;54:B207–B218. 10362000 10.1093/gerona/54.5.b207

[135] Frontera WR , Hughes VA , Fielding RA , Fiatarone MA , Evans WJ , Roubenoff R . Aging of skeletal muscle: a 12‐yr longitudinal study. J Appl Physiol (1985). 2000;88:1321–1326. 10749826 10.1152/jappl.2000.88.4.1321

[136] Trappe S , Gallagher P , Harber M , Carrithers J , Fluckey J , Trappe T . Single muscle fibre contractile properties in young and old men and women. The Journal of physiology. 2003;552:47–58. 12837929 10.1113/jphysiol.2003.044966 PMC2343327

[137] Wolfson L , Wei X , Hall CB , Panzer V , Wakefield D , Benson RR , et al. Accrual of MRI white matter abnormalities in elderly with normal and impaired mobility. J Neurol Sci. 2005;232:23–27. 15850578 10.1016/j.jns.2004.12.017

[138] Guralnik JM , Ferrucci L , Simonsick EM , Salive ME , Wallace RB . Lower‐extremity function in persons over the age of 70 years as a predictor of subsequent disability. N Engl J Med. 1995;332:556–562. 7838189 10.1056/NEJM199503023320902 PMC9828188

[139] Wright AA , Cook CE , Baxter GD , Garcia J , Abbott JH . Relationship between the Western Ontario and McMaster Universities Osteoarthritis Index Physical Function Subscale and physical performance measures in patients with hip osteoarthritis. Arch Phys Med Rehabil. 2010;91:1558–1564. 20875514 10.1016/j.apmr.2010.07.016

[140] Evans WJ , Paolisso G , Abbatecola AM , Corsonello A , Bustacchini S , Strollo F , et al. Frailty and muscle metabolism dysregulation in the elderly. Biogerontology. 2010;11:527–536. 20683658 10.1007/s10522-010-9297-0

[141] Inzitari M , Pozzi C , Ferrucci L , Chiarantini D , Rinaldi LA , Baccini M , et al. Subtle neurological abnormalities as risk factors for cognitive and functional decline, cerebrovascular events, and mortality in older community‐dwelling adults. Arch Intern Med. 2008;168:1270–1276. 18574083 10.1001/archinte.168.12.1270 PMC4642714

[142] Corsonello A , Lattanzio F , Pedone C , Garasto S , Laino I , Bustacchini S , et al. Prognostic significance of the short physical performance battery in older patients discharged from acute care hospitals. Rejuvenation research. 2012;15:41–48. 22004280 10.1089/rej.2011.1215 PMC3283437

[143] Gawel J , Vengrow D , Collins J , Brown S , Buchanan A , Cook C . The short physical performance battery as a predictor for long term disability or institutionalization in the community dwelling population aged 65 years old or older. Physical Therapy Reviews. 2012;17:37–44.

[144] Taekema DG , Gussekloo J , Westendorp RG , De Craen AJ , Maier AB . Predicting survival in oldest old people. Am J Med. 2012;125:1188–94. e1. 23017181 10.1016/j.amjmed.2012.01.034

[145] Cesari M , Kritchevsky SB , Penninx BW , Nicklas BJ , Simonsick EM , Newman AB , et al. Prognostic value of usual gait speed in well‐functioning older people—results from the Health, Aging and Body Composition Study. J Am Geriatr Soc. 2005;53:1675–1680. 16181165 10.1111/j.1532-5415.2005.53501.x

[146] Woolley D , Studenski S , Perera S , Rogers N . Feasibility and reproducibility of walking speed as a geriatric vitalsign incommunity practice. J Am Geriatr Soc. 2004;52:S195.

[147] Bahat G , Tufan A , Kilic C , Karan MA , Cruz‐Jentoft AJ . Prevalence of sarcopenia and its components in community‐dwelling outpatient older adults and their relation with functionality. Aging Male. 2018;1–7. 10.1080/13685538.2018.1511976 30290756

[148] Perera S , Patel KV , Rosano C , Rubin SM , Satterfield S , Harris T , et al. Gait speed predicts incident disability: a pooled analysis. J Gerontol A Biol Sci Med Sci. 2016;71:63–71. 26297942 10.1093/gerona/glv126 PMC4715231

[149] Perera S , Studenski S , Newman A , Simonsick E , Harris T , Schwartz A , et al. Are estimates of meaningful decline in mobility performance consistent among clinically important subgroups? (Health ABC Study). J Gerontol A Biol Sci Med Sci. 2014;69:1260–1268. 24615070 10.1093/gerona/glu033 PMC4172035

[150] Cummings SR , Studenski S , Ferrucci L . A diagnosis of dismobility—giving mobility clinical visibility: a Mobility Working Group recommendation. JAMA. 2014;311:2061–2062. 24763978 10.1001/jama.2014.3033 PMC5012417

[151] Clark DO , Stump TE , Hui SL , Wolinsky FD . Predictors of mobility and basic ADL difficulty among adults aged 70 years and older. J Aging Health. 1998;10:422–440. 10346693 10.1177/089826439801000402

[152] Manchester D , Woollacott M , Zederbauer‐Hylton N , Marin O . Visual, vestibular and somatosensory contributions to balance control in the older adult. J Gerontol. 1989;44:M118–M127. 2786896 10.1093/geronj/44.4.m118

[153] Pasma J , Engelhart D , Schouten A , Van der Kooij H , Maier A , Meskers C . Impaired standing balance: the clinical need for closing the loop. Neuroscience. 2014;267:157–165. 24613719 10.1016/j.neuroscience.2014.02.030

[154] Vellas BJ , Wayne SJ , Romero L , Baumgartner RN , Rubenstein LZ , Garry PJ . One‐leg balance is an important predictor of injurious falls in older persons. J Am Geriatr Soc. 1997;45:735–738. 9180669 10.1111/j.1532-5415.1997.tb01479.x

[155] Gill TM , Williams CS , Tinetti ME . Assessing risk for the onset of functional dependence among older adults: the role of physical performance. J Am Geriatr Soc. 1995;43:603–609. 7775716 10.1111/j.1532-5415.1995.tb07192.x

[156] Podsiadlo D , Richardson S . The timed “Up & Go”: a test of basic functional mobility for frail elderly persons. J Am Geriatr Soc. 1991;39:142–148. 1991946 10.1111/j.1532-5415.1991.tb01616.x

[157] Bohannon RW . Reference values for the timed up and go test: a descriptive meta‐analysis. J Geriatr Phys Ther. 2006;29:64–68. 16914068 10.1519/00139143-200608000-00004

[158] Lin MR , Hwang HF , Hu MH , Wu HDI , Wang YW , Huang FC . Psychometric comparisons of the timed up and go, one‐leg stand, functional reach, and Tinetti balance measures in community‐dwelling older people. Journal of the American Geriatrics Society. 2004;52:1343–1348. 15271124 10.1111/j.1532-5415.2004.52366.x

[159] Jones CJ , Rikli RE , Beam WC . A 30‐s chair‐stand test as a measure of lower body strength in community‐residing older adults. Res Q Exerc Sport. 1999;70:113–119. 10380242 10.1080/02701367.1999.10608028

[160] Carriere I , Colvez A , Favier F , Jeandel C , Blain H , group Es . Hierarchical components of physical frailty predicted incidence of dependency in a cohort of elderly women. J Clin Epidemiol. 2005;58:1180–1187. 16223662 10.1016/j.jclinepi.2005.02.018

[161] von Haehling S , Morley JE , Coats AJ , Anker SD . Ethical guidelines for publishing in the *Journal of Cachexia, Sarcopenia and Muscle*: update 2017. J Cachexia Sarcopenia Muscle. 2017;8:1081–1083. 29098794 10.1002/jcsm.12261 PMC5700441

