# Supplementary material for: Muscle mass, strength, and physical performance predicting activities of daily living: a meta‐analysis
Source: J Cachexia Sarcopenia Muscle. 2019 Dec 1;11(1):3–25. doi: 10.1002/jcsm.12502 (PMC7015244; doi:10.1002/jcsm.12502)
Supplement: Supplementary file 1 — Table S1. Search Strategy [file JCSM-11-3-s001.docx]

**Supplementary Table 1. Search Strategy**

1. **MEDLINE**

Databases(s): **Ovid MEDLINE(R) Epub Ahead of Print, In-Process & Other Non-Indexed Citations, Ovid Medline(R) Daily and Ovid Medline(R)** 1946 to Present

| # | Searches | Results |
| --- | --- | --- |
| 1 | ((muscle or muscles or muscular) adj1 (mass or weakness or weak or atroph* or wasting or wasted or sarcopeni*)).mp. [mp=title, abstract, original title, name of substance word, subject heading word, keyword heading word, protocol supplementary concept word, rare disease supplementary concept word, unique identifier, synonyms] | 54466 |
| 2 | ("fat free mass" or "lean mass").mp. [mp=title, abstract, original title, name of substance word, subject heading word, keyword heading word, protocol supplementary concept word, rare disease supplementary concept word, unique identifier, synonyms] | 10826 |
| 3 | 1 or 2 | 63866 |
| 4 | ((muscle* or muscular) adj3 strength*).mp. [mp=title, abstract, original title, name of substance word, subject heading word, keyword heading word, protocol supplementary concept word, rare disease supplementary concept word, unique identifier, synonyms] | 34195 |
| 5 | ((Physical adj2 (performanc* or mobilit* or fitness* or enduranc*)) or (Walk* adj2 test*) or ((gait* or walk*) and speed) or (stand* adj2 (balanc* or test*)) or (balanc* adj2 (test* or impairment*)) or ((Time* adj1 (up adj2 go test*)) or up-and-go or sit-to-stand or chair-to-stand) or comprehensive geriatric assessment* or (geriatric evaluation adj2 management*)).mp. [mp=title, abstract, original title, name of substance word, subject heading word, keyword heading word, protocol supplementary concept word, rare disease supplementary concept word, unique identifier, synonyms] | 105983 |
| 6 | 3 or 4 or 5 | 186950 |
| 7 | (Activit* of daily living or adl or iadl or bathing or dressing or toileting or (transfer* adj3 (chairs or chair or bed or beds)) or continence).mp. [mp=title, abstract, original title, name of substance word, subject heading word, keyword heading word, protocol supplementary concept word, rare disease supplementary concept word, unique identifier, synonyms] | 110932 |
| 8 | ((disabil* adj3 function) or functional decline).mp. [mp=title, abstract, original title, name of substance word, subject heading word, keyword heading word, protocol supplementary concept word, rare disease supplementary concept word, unique identifier, synonyms] | 15819 |
| 9 | 7 or 8 | 123041 |
| 10 | 6 and 9 | 7962 |
| 11 | limit 12 to ("middle aged (45 plus years)" or "all aged (65 and over)") | 5795 |
| 12 | (elderly or ((old or older or aged) adj (person* or patient* or people or male or female or males or females or men or women or individual* or population)) or elder or geriatric*).mp. [mp=title, abstract, original title, name of substance word, subject heading word, keyword heading word, protocol supplementary concept word, rare disease supplementary concept word, unique identifier, synonyms] | 550055 |
| 13 | 10 not 11 | 2167 |
| 14 | 12 and 13 | 303 |
| 15 | 11 or 14 | 6098 |
| 16 | (case report* or editorial or letter).pt. | 3133800 |
| 17 | 15 not 16 | 5946 |
| 18 | limit 17 to English language | 5540 |

1. **EMBASE**

Databases(s): **Embase Classic + Embase** 1947 to Present

| # | Searches | Results |
| --- | --- | --- |
| 1 | ((muscle or muscles or muscular) adj1 (mass or weakness or weak or atroph* or wasting or wasted or sarcopeni*)).mp. [mp=title, abstract, original title, name of substance word, subject heading word, keyword heading word, protocol supplementary concept word, rare disease supplementary concept word, unique identifier, synonyms] | 114729 |
| 2 | ("fat free mass" or "lean mass").mp. [mp=title, abstract, original title, name of substance word, subject heading word, keyword heading word, protocol supplementary concept word, rare disease supplementary concept word, unique identifier, synonyms] | 15924 |
| 3 | 1 or 2 | 127912 |
| 4 | ((muscle* or muscular) adj3 strength*).mp. [mp=title, abstract, original title, name of substance word, subject heading word, keyword heading word, protocol supplementary concept word, rare disease supplementary concept word, unique identifier, synonyms] | 64189 |
| 5 | ((Physical adj2 (performanc* or mobilit* or fitness* or enduranc*)) or (Walk* adj2 test*) or ((gait* or walk*) and speed) or (stand* adj2 (balanc* or test*)) or (balanc* adj2 (test* or impairment*)) or ((Time* adj1 (up adj2 go test*)) or up-and-go or sit-to-stand or chair-to-stand) or comprehensive geriatric assessment* or (geriatric evaluation adj2 management*)).mp. [mp=title, abstract, original title, name of substance word, subject heading word, keyword heading word, protocol supplementary concept word, rare disease supplementary concept word, unique identifier, synonyms] | 123717 |
| 6 | 3 or 4 or 5 | 288999 |
| 7 | (Activit* of daily living or adl or iadl or bathing or dressing or toileting or (transfer* adj3 (chairs or chair or bed or beds)) or continence).mp. [mp=title, abstract, original title, name of substance word, subject heading word, keyword heading word, protocol supplementary concept word, rare disease supplementary concept word, unique identifier, synonyms] | 110480 |
| 8 | ((disabil* adj3 function) or functional decline).mp. [mp=title, abstract, original title, name of substance word, subject heading word, keyword heading word, protocol supplementary concept word, rare disease supplementary concept word, unique identifier, synonyms] | 23423 |
| 9 | 7 or 8 | 131245 |
| 10 | 6 and 9 | 9021 |
| 11 | (elderly or ((old or older or aged) adj (person* or patient* or people or male or female or males or females or men or women or individual* or population)) or elder or geriatric*).mp. [mp=title, abstract, original title, name of substance word, subject heading word, keyword heading word, protocol supplementary concept word, rare disease supplementary concept word, unique identifier, synonyms] | 921503 |
| 12 | limit 10 to aged <65+ years> | 4349 |
| 13 | 10 and 11 | 3692 |
| 14 | 12 or 13 | 5166 |
| 15 | (case report* or editorial or letter or conference abstract).pt. | 4622854 |
| 16 | 14 not 15 | 4001 |
| 17 | limit 16 to english language | 3733 |

1. **COCHRANE**

Database(s): **EBM Reviews - Cochrane Central Register of Controlled Trials**

| # | Searches | Results |
| --- | --- | --- |
| 1 | ((muscle or muscles or muscular) adj1 (mass or weakness or weak or atroph* or wasting or wasted or sarcopeni*)).mp. [mp=title, abstract, original title, name of substance word, subject heading word, keyword heading word, protocol supplementary concept word, rare disease supplementary concept word, unique identifier, synonyms] | 4398 |
| 2 | ("fat free mass" or "lean mass").mp. [mp=title, abstract, original title, name of substance word, subject heading word, keyword heading word, protocol supplementary concept word, rare disease supplementary concept word, unique identifier, synonyms] | 2117 |
| 3 | 1 or 2 | 6151 |
| 4 | ((muscle* or muscular) adj3 strength*).mp. [mp=title, abstract, original title, name of substance word, subject heading word, keyword heading word, protocol supplementary concept word, rare disease supplementary concept word, unique identifier, synonyms] | 10255 |
| 5 | ((Physical adj2 (performanc* or mobilit* or fitness* or enduranc*)) or (Walk* adj2 test*) or ((gait* or walk*) and speed) or (stand* adj2 (balanc* or test*)) or (balanc* adj2 (test* or impairment*)) or ((Time* adj1 (up adj2 go test*)) or up-and-go or sit-to-stand or chair-to-stand) or comprehensive geriatric assessment* or (geriatric evaluation adj2 management*)).mp. [mp=title, abstract, original title, name of substance word, subject heading word, keyword heading word, protocol supplementary concept word, rare disease supplementary concept word, unique identifier, synonyms] | 23198 |
| 6 | 3 or 4 or 5 | 34428 |
| 7 | (Activit* of daily living or adl or iadl or bathing or dressing or toileting or (transfer* adj3 (chairs or chair or bed or beds)) or continence).mp. [mp=title, abstract, original title, name of substance word, subject heading word, keyword heading word, protocol supplementary concept word, rare disease supplementary concept word, unique identifier, synonyms] | 13152 |
| 8 | ((disabil* adj3 function) or functional decline).mp. [mp=title, abstract, original title, name of substance word, subject heading word, keyword heading word, protocol supplementary concept word, rare disease supplementary concept word, unique identifier, synonyms] | 2899 |
| 9 | 7 or 8 | 15629 |
| 10 | 6 and 9 | 1986 |
| 11 | (elderly or ((old or older or aged) adj (person* or patient* or people or male or female or males or females or men or women or individual* or population)) or elder or geriatric*).mp. [mp=title, abstract, original title, name of substance word, subject heading word, keyword heading word, protocol supplementary concept word, rare disease supplementary concept word, unique identifier, synonyms] | 204997 |
| 12 | 10 and 11 | 1117 |
| 13 | limit 12 to english language | 1043 |

1. **CINAHL Complete**

| # | Searches | Search Options | Results |
| --- | --- | --- | --- |
| 1 | ((muscle or muscles or muscular) W1 (mass or weakness or weak or atroph* or wasting or wasted or sarcopeni*)) OR ("fat free mass" or "lean muscle mass") | Search modes – Boolean/Phase | 15018 |
| 2 | ((muscle* or uscular) W3 strength*) | Search modes – Boolean/Phase | 29455 |
| 3 | ((Physical W2 (performanc* or mobilit* or fitness* or enduranc*)) or (Walk* W2 test*) or ((gait* or walk*) and speed) or (stand* W2 (balanc* or test*)) or (balanc* W2 (test* or impairment*)) or ((Time* W1 (up W2 go test*)) or up-and-go or sit-to-stand or chair-to-stand) or comprehensive geriatric assessment* or (geriatric evaluation W2 management*)) | Search modes – Boolean/Phase | 49783 |
| 4 | S1 or S2 or S3 | Search modes – Boolean/Phase | 83603 |
| 5 | (Activit* of daily living or adl or iadl or bathing or dressing or toileting or (transfer* W3 (chairs or chair or bed or beds)) or continence) | Search modes – Boolean/Phase | 56419 |
| 6 | ((disabil* W3 function) or functional decline) | Search modes – Boolean/Phase | 2742 |
| 7 | S5 OR S6 | Search modes – Boolean/Phase | 58399 |
| 8 | S4 AND S7 | Search modes – Boolean/Phase | 4576 |
| 9 | Elderly or aged or older or elder or geriatric | Search modes – Boolean/Phase | 829703 |
| 10 | S8 AND S9 | Search modes – Boolean/Phase | 3148 |
| 11 | S10 | Limiters – English Language; Age Groups: Aged: 65+ years | 2634 |
