# Supplementary material for: Muscle mass, strength, and physical performance predicting activities of daily living: a meta‐analysis
Source: J Cachexia Sarcopenia Muscle. 2019 Dec 1;11(1):3–25. doi: 10.1002/jcsm.12502 (PMC7015244; doi:10.1002/jcsm.12502)
Supplement: Supplementary file 2 — Data S2. Newcastle–Ottawa Scale [file JCSM-11-3-s002.docx]

**Supplementary Material 2. Newcastle-Ottawa Scale**

**Newcastle-Ottawa Scale (NOS) – Cohort Studies**

Note: A study can be awarded a maximum of one star for each numbered item within the Selection and Outcome categories. A maximum of two stars can be given for Comparability

**Selection**

1. Representativeness of the exposed cohort
   1. Truly representative of the average older population aged 65 years and older at follow up *
   2. Not representative or no description
2. In the case of cohort that was dichotomised: subjects were the recruited from the same cohort
   1. Yes *
   2. No
3. Ascertainment of exposure: how was muscle mass, muscle strength and physical performance measured
   1. Muscle mass was measured by one of: BIA, DXA, CT or MRI. Muscle strength was measured by one of: HGS, LL or UL. Physical performance was measured: objectively, by a healthcare professional or measured using device. *
   2. Self-reported retrospectively
   3. Unclear or not reported

**Comparability**

1. Comparability of cohorts on the basis of the design or analysis
   1. Study controls for: age and/or sex *
   2. Study controls for: other factors *
   3. Study does not control for confounders
   4. Study does not perform any statistical analysis or not reported

**Outcome**

1. Assessment of activities of daily living or instrumental activities of daily living outcome
   1. Validated method *
   2. Designed own questionnaire or survey or method *
   3. Not reported or described
2. Was follow-up long enough for outcome to occur
   1. Yes, ≥ 3 months *
   2. No, < 3 months
   3. Not reported
3. Adequacy of follow up of cohorts
   1. Complete follow up – all subjects accounted for *
   2. Subjects lost to follow up unlikely to introduce bias – number lost ≤ 20% or description of those lost suggested no different from those followed *
   3. Follow up rate less than 80% and no description of those lost
   4. Not described or not applicable

* = 1 star
