# Supplementary material for: Muscle mass, strength, and physical performance predicting activities of daily living: a meta‐analysis
Source: J Cachexia Sarcopenia Muscle. 2019 Dec 1;11(1):3–25. doi: 10.1002/jcsm.12502 (PMC7015244; doi:10.1002/jcsm.12502)
Supplement: Supplementary file 3 — Table S3. Breakdown of the ADL components in articles that created their own questionnaire [file JCSM-11-3-s003.docx]

**Supplementary Table 3. Breakdown of the ADL components in articles that created their own questionnaire**

| **First author (year)[ref]** | **D** | **B** | **F** | **W** | **Tr** | **To** | **C** | **G** | **S** | **O** |
| --- | --- | --- | --- | --- | --- | --- | --- | --- | --- | --- |
| Beloosesky (2009)[40] | x | x | x |  |  | x | x | x |  |  |
| Broadwin (2001)[27] | x |  |  | x |  |  |  |  |  | x |
| Chaudhry (2010)[42] | x | x | x | x | x | x |  |  |  |  |
| Cooper (2011)[76] | x | x |  | x |  |  |  | x | x |  |
| Donoghue (2014)[79] | x | x | x |  | x | x | x |  |  |  |
| Duchowny (2018)[46] | x | x | x | x | x | x |  |  |  |  |
| Fantin (2007)[30] | x | x | x |  | x |  |  |  |  |  |
| Femia (1997)[47] | x | x | x |  | x | x |  | x |  |  |
| Fujiwara (2016)[80] | x | x | x | x |  |  | x |  |  |  |
| Gill (2009)[49] | x | x |  | x | x |  |  |  |  |  |
| Guralnik (2000)[82] |  | x |  | x | x | x |  |  |  |  |
| Heiland (2016)[84] | x | x | x |  | x |  |  |  |  |  |
| Ishizaki (2000)[50] | x | x | x | x |  |  | x |  |  |  |
| Jonkman (2018)[16] | x |  |  |  | x |  |  |  |  |  |
| Legrand (2014)[53] | x |  |  | x | x |  |  | x | x | x |
| Lopez-Teros (2014)[54] | NR |  |  |  |  |  |  |  |  |  |
| Minneci (2015)[56] | x | x | x |  | x | x | x |  |  |  |
| Onder (2005)[58] | x | x | x |  |  | x |  |  |  |  |
| Ostir (1998)[89] |  | x |  | x |  | x |  |  |  |  |
| Rantanen (1999)[60] | x | x | x |  |  | x |  |  |  | x |
| Rantanen (2002)[61] | x | x |  | x | x | x |  |  |  |  |
| Rothman (2008)[63] | NR |  |  |  |  |  |  |  |  |  |
| Sakamoto (2016)[93] | x | x | x | x |  | x |  | x | x |  |
| Sanchez-Martinez (2016)[94] |  |  |  | x |  |  |  |  | x |  |
| Sarkisian (2000)[64] | x | x | x |  | x |  |  |  |  | x |
| Shinkai (2000)[67] | x | x |  | x |  |  | x |  |  |  |
| Shinkai (2003)[68] | x | x | x | x |  |  | x |  |  |  |
| Stenholm (2014)[99] | x | x | x | x | x | x |  |  |  |  |
| Volpato (2011)[103] | x | x | x |  | x | x |  | x |  |  |
| Zhang (2013)[104] | x | x | x |  | x | x |  |  |  |  |
| Total | 25 | 24 | 18 | 16 | 16 | 15 | 7 | 6 | 4 | 4 |

**D:** dressing; **B:** bathing; **F:** feeding; **W:** walking; **Tr:** transferring; **To:** toilet; **C:** continence; **G:** grooming; **S:** stairs; **O:** other(s); **NR:** not reported
